# Are neurodegenerative diseases associated with an increased risk of inflammatory bowel disease? A two-sample Mendelian randomization study

**DOI:** 10.3389/fimmu.2022.956005

**Published:** 2022-09-08

**Authors:** Guanghui Cui, Shaojie Li, Hui Ye, Yao Yang, Qiuyue Huang, Yingming Chu, Zongming Shi, Xuezhi Zhang

**Affiliations:** ^1^ Department of Integrated Traditional Chinese and Western Medicine, Peking University First Hospital; Institute of Integrated Traditional Chinese and Western Medicine, Peking University, Beijing, China; ^2^ School of Public Health, Peking University, Beijing, China

**Keywords:** inflammatory bowel disease, ulcerative colitis, crohn’s disease, parkinson’s disease, alzheimer’s disease, Mendelian randomization

## Abstract

**Background:**

Several studies have shown that neurodegenerative diseases (e.g., Parkinson’s disease [PD] and Alzheimer’s disease [AD]) are associated with inflammatory bowel disease (IBD), but the causality and direction of their associations remain unclear. Mendelian randomization (MR) studies have explored the causal effects of IBD on PD and AD. However, only a few studies examined this reverse association. Thus, this study aimed to explore whether there are causal associations of genetically predicted PD and AD with IBD, using a two-sample MR study.

**Methods:**

Summary statistics for IBD, ulcerative colitis (UC), and Crohn’s disease (CD) were derived from a genome-wide association study (GWAS) meta-analysis, which included the International IBD Genetics Consortium and the UK IBD Genetics Consortium (n=59,957). Genetic variants associated with the largest meta-analysis of GWAS of PD (n=1,474,097) and AD (n=455,258) were used as instrumental variables. We used multiple methods, including inverse variance weighted (IVW), weighted median (WM), MR-Egger regression, weighted mode, and Robust Adjusted Profile Score (RAPS) methods, to estimate the effects of genetically predicted PD and AD on IBD. To confirm the validity of the analysis, we also evaluated the pleiotropic effects, heterogeneity, and leave-one-out sensitivity analysis that drive causal associations.

**Results:**

The results of the IVW method, WM, and RAPS showed that genetically predicted PD was significantly associated with an increased risk of UC (odds ratio [*OR*]_IVW_=1.068, *OR*_WM_=1.107, *OR*_RAPS_=1.069, all *P*<0.05). Additionally, we found that there were significant associations of genetically predicted PD with CD (*OR*_IVW_=1.064, *OR*_RAPS_=1.065, all *P*<0.05) and IBD (*OR*_IVW_=1.062, *OR*_RAPS_=1.063, all *P*<0.05) using the IVW method and RAPS. However, there was no significant causal evidence of genetically predicted AD in IBD, UC, or CD among all MR methods. In all MR analyses, there were no horizontal pleiotropy (all *P*>0.05), or statistical heterogeneity. The sensitivity analysis results of the leave-one-out sensitivity analysis showed that the causal effect estimations of genetically predicted PD and AD on IBD were robust.

**Conclusions:**

Our MR study corroborated a causal association between genetically predicted PD and IBD but did not support a causal effect of genetically predicted AD on IBD. More animal experiments or population-based observational studies are required to clarify the underlying mechanisms of PD and IBD.

## Introduction

Inflammatory bowel disease (IBD) is a chronic intestinal disorder caused by genetic susceptibility, abnormal intestinal mucosal immune system function, microbiota imbalance, and other factors (1). The two typical subtypes are Crohn’s disease (CD) and ulcerative colitis (UC). CD can affect any part of the intestines in a noncontiguous transmural inflammation manner and can lead to intestinal wall thickening, narrowing, fibrosis, abscesses, and fistulas (2). In contrast, UC is usually limited to the mucosal surface, leaving the deeper layers of the colon unaffected and extending proximally in a continuous pattern, which can lead to ulcerations and bleeding (1). IBD not only reduces patients’ quality of life significantly but also creates a serious economic and medical burden to the society due to its high prevalence (3). It is also accompanied by various complications or extra-intestinal manifestations (4). Therefore, it is imperative to explore the risk factors and pathogenesis of IBD to achieve improved prevention and control.

Increasing evidence has shown that the gut-brain axis is pivotal in connecting neurological and gastrointestinal diseases. This communication system, driven by neural, hormonal, immunological, microbial, metabolic, and other signals, is expected to provide a new perspective for explaining the relationship between IBD and neurodegenerative diseases (5). For example, a cohort study based on the Korean population suggested that patients with IBD had a higher risk of neurodegenerative diseases (Parkinson’s disease [PD] and Alzheimer’s disease [AD]) than non-IBD controls (6). Cohort studies from Denmark (7) and Taiwan (8) demonstrated potential relationships between IBD, PD, and dementia. However, the aforementioned studies were all observational, and most of them were based on administrative data initially collected for non-statistical reasons. There were various measurement errors, underlying biases (e.g., surveillance bias), and confounding factors (e.g., history of unhealthy habits, disease occurrence, development, and medication) (9), which may reverse causality and make it difficult to distinguish the real causes and consequences.

Recently, Mendelian randomization (MR), a method of causal inference using genetic variants, has provided evidence based on Mendel’s second law (the random assortment of alleles during gamete formation and conception) to explore the causality between exposure and outcome using single-nucleotide polymorphisms (SNPs) that are associated with exposure but independent of confounding factors linked to the selected exposure and outcome as instrumental variables (IVs) (10, 11). Freuer et al. conducted an MR study of the association between IBD and PD and reported that genetically predicted IBD was not associated with an increased risk of PD (12). Unfortunately, their analysis was unidirectional, which means that the question of whether PD can increase the risk of IBD remains unanswered. Recently, several studies have suggested that there may be bidirectional associations between IBD and neurodegenerative diseases (9, 13–15). However, these studies mainly focused on the association between IBD and increased risks of neurodegenerative diseases (9). Only a few observational studies have explored the reverse association, which is whether neurodegenerative diseases will also increase the risk of IBD. This may be the case because the onset age of most IBDs is generally considered to be earlier than that of neurodegenerative diseases. However, it cannot be ignored that the incidence and prevalence of elderly-onset IBD have been increasing in recent years (16). Cohort studies from different countries and regions, such as France (17), Hong Kong (18), Sweden (19), and Canada (20), have shown that 7.5%–23.2% of newly diagnosed individuals with IBD are older than 60 years. With the increasing proportion of older adults in the population, the incidence and prevalence of elderly-onset IBD are expected to increase further (21, 22). Therefore, attention to the effect of neurodegenerative diseases on IBD may provide a new concept for IBD prevention in the field of aging health. Based on the above epidemiological evidence, there is reason to explore the impact of neurodegenerative diseases on IBD.

In addition, a previous meta-analysis of observational studies also reported that existing studies lacked a reverse causal association between IBD and PD (23). To the best of our knowledge, only two case-control studies based on health care program or hospital registration data have explored the association between PD and the risk of IBD (24, 25), but the results were inconsistent. A previous observational study based on health care program data in the United States showed that patients with PD had a lower risk of IBD than those without PD (24), whereas a study based on Danish hospital registration data suggested that there was no significant association between IBD and PD (25). Note that the above two studies were cross-sectional, prohibiting any inference on the causal relationship between IBD and PD. In addition, an MR study found that genetically predicted IBD is associated with a reduced risk of AD (26). Similar to the study on the association between PD and IBD, no study has explored the reverse causal effects, that is, whether AD increases the risk of IBD. Through a bidirectional MR design, Yeung et al. found that none of the genetically predicted 41 systemic inflammatory regulators were associated with the risk of AD. Conversely, AD was associated with five systemic inflammatory regulators, which implied that genetically predicted systemic inflammation may be a downstream effect of AD or a consequence of comorbidity factors (27). As inflammation is the core pathological mechanism of IBD, we should further verify whether AD leads to an increased risk of IBD.

Finally, it should be pointed out that the biological processes involved in the gut-brain axis are bidirectional interactions, but the role of the top-down pathway in the association between neurodegenerative diseases and IBD has not been thoroughly investigated. It has not been studied whether the enteric nervous system, neuro-immune crosstalk, and resulting IBD are affected by the etiology of these neurodegenerative diseases (28). Considering the existing research gaps, it is necessary to further explore the reverse causal association between PD, AD, and IBD to clarify the direction of causality, which would help to deepen our understanding of the bidirectional association between IBD and neurodegenerative diseases. Given the potential harm of IBD to patients’ quality of life (29), it would also be helpful to establish a scientific basis for the prevention of IBD and the improvement of quality of life in patients with neurodegenerative diseases in the future. Therefore, this study used a two-sample MR analysis to explore the causal associations of genetically predicted PD and AD with IBD.

## Materials and methods

### Study design

**Figure 1:**
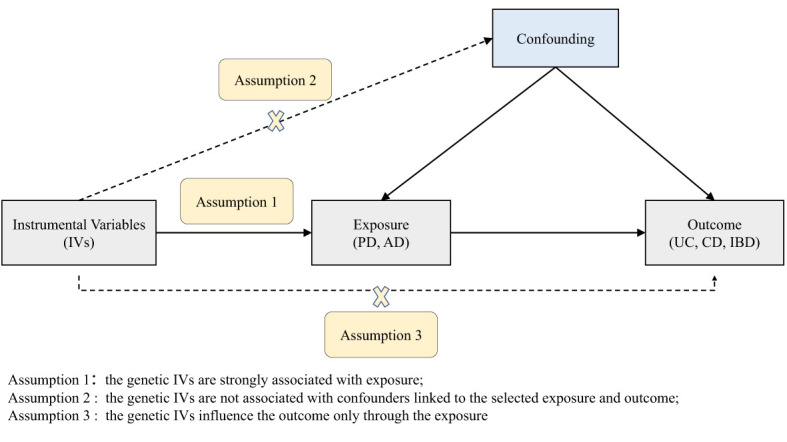
Diagram of the two-sample Mendelian randomization study for the associations of PD and AD with UC, CD, and IBD. PD, Parkinson’s disease; AD, Alzheimer’s disease; IBD, inflammatory bowel disease; UC, ulcerative colitis; CD, Crohn’s disease.

According to the basic principles and core assumptions of MR, three assumptions were met in this study (**Figure 1**) (1): the genetic IVs are strongly associated with PD/AD; (2) the genetic IVs are not associated with confounders linked to the PD/AD and IBD; (3) the genetic IVs influence IBD only through the PD/AD. The datasets used in our study are publicly available and received ethical approval and informed consent prior to implementation. Therefore, our study did not require additional ethical approval or informed consent.

### Data sources

Summary statistics for IBD, UC, and CD were derived from a genome-wide association study (GWAS) meta-analysis that included the International IBD Genetics Consortium and UK IBD Genetics Consortium (n=59,957). This study included IBD samples (n=25,042 cases, 34,915 controls), UC samples (n=12,366 cases, 33,609 controls), and CD samples (n=12,194 cases, 28,072 controls) of European ancestry (30).

Genetic variants associated with PD were obtained from the largest GWAS meta-analysis performed by the International Parkinson’s Disease Genomics Consortium, including 56,306 PD cases and 1,417,791 controls (31). We selected genome-wide significant SNPs as IVs for PD.

Genetic variants associated with AD were obtained from a GWAS meta-analysis of AD (32). This study included 71,880 cases and 383,378 controls of European ancestry from the International Genomics of Alzheimer’s Project, Alzheimer’s Disease Sequencing Project, Alzheimer’s Disease Working Group of the Psychiatric Genomics Consortium, and UK Biobank (32).

### Genetic IVs

We strictly conducted a series of quality control techniques to filter eligible genetic instruments. First, we selected SNPs that were strongly associated with PD and AD (*P*<5×10^-8^) at the genome-wide significance level. The SNPs identified by two GWAS explained 22.0% and 7.1% of the heritability in PD and AD. Second, to exclude the variants in strong linkage disequilibrium (LD) and ensure the independence of each SNP, we performed the clumping procedure with standard parameter SNPs (*R* ^2^<0.001, window size=10,000 kb). The SNPs among pairings with an LD *R* ^2^ value greater than the specified threshold with a lower *P*-value were retained. SNPs with a minor allele frequency <0.01 were eliminated. Third, the SNPs associated with IBD (*P*<5×10^-6^) were excluded by screening the GWAS Catalog (33). Finally, ambiguous SNPs with non-concordant alleles (e.g., A/G vs. A/C) and palindromic SNPs (e.g., A/T or G/C) were excluded from the process of harmonizing the exposure and outcome datasets.

### Statistical analysis

Before MR analysis, we calculated the *F* statistics (*F*=*R* ^2^(n-k-1)/k(1-*R* ^2^)) of the PD and AD IVs to determine whether there was a weak IV bias (34). Among them, *R* ^2^ represents the variance of exposure explained by the IV, n is the sample size of the GWAS, and k is the number of IVs. The calculated result of *F* statistics <10 indicates a weak IV bias (35).

We used multiple methods, including inverse variance weighted (IVW), MR-Egger regression, weighted median (WM), weighted mode, and Robust Adjusted Profile Score (RAPS) methods, to estimate the effect of exposure on outcome susceptibility. The IVW method summarizes the weighted average of Wald ratio estimates of the causal effects for each variant (36), which can provide the most precise estimated results when all selected SNPs are valid IVs. The MR-Egger regression methods perform weighted linear regression analysis under the assumption that the associations between genetic variants and exposure are independent of the direct effects of genetic variants on the outcome (InSIDE assumption). Even if all genetic variants are invalid IVs, the MR-Egger test yields a valid test of the null causal hypothesis and a consistent causal estimate (37). However, this method exhibits low statistical precision and is susceptible to outlying genetic variants (38). The WM method required at least 50% of the weight of valid IVs. It is significantly and consistently more precise than the MR-Egger method and more robust to violations of causal effects (39). The weighted mode method estimates the causal effect of the subset with the largest number of SNPs by clustering SNPs into groups according to the similarity of causal effects. It has less power to detect causal effects than IVW and WM methods (40). However, in some cases, this method presents the characteristics of less bias and lower type-I error rates (40). RAPS considers the measurement error in SNP-exposure effects and the biases caused by weak instruments, and is robust for alleviating pleiotropy (41).

The intercept term of the MR-Egger regression test was used to estimate the possibility of horizontal pleiotropy. Additionally, we conducted the MR-PRESSO global test to evaluate the existence of horizontal pleiotropy and deleted outlier variants (42). Heterogeneity was quantified by calculating Cochran’s Q statistic. We also conducted a leave-one-out sensitivity analysis. Alpha <0.05 was set as the two-sided threshold for statistical significance.

All statistical analyses were performed using the “TwoSampleMR” package (https://github.com/MRCIEU/TwoSampleMR) (43) and the “MR-PRESSO” package in R (The R Project for Statistical Computing), version 4.2.0.

## Results

### Selection of IVs

Through the aforementioned series of screening processes, 54 and 23 independent SNPs were selected as the IVs of PD and AD, respectively. The *F* statistics of all IVs were >10, indicating a slight possibility of a weak IV bias. Detailed information about all the IVs is provided in **Supplementary Tables S1**, **S2**.

### Causal effects of PD on IBD

**Figure 2:**
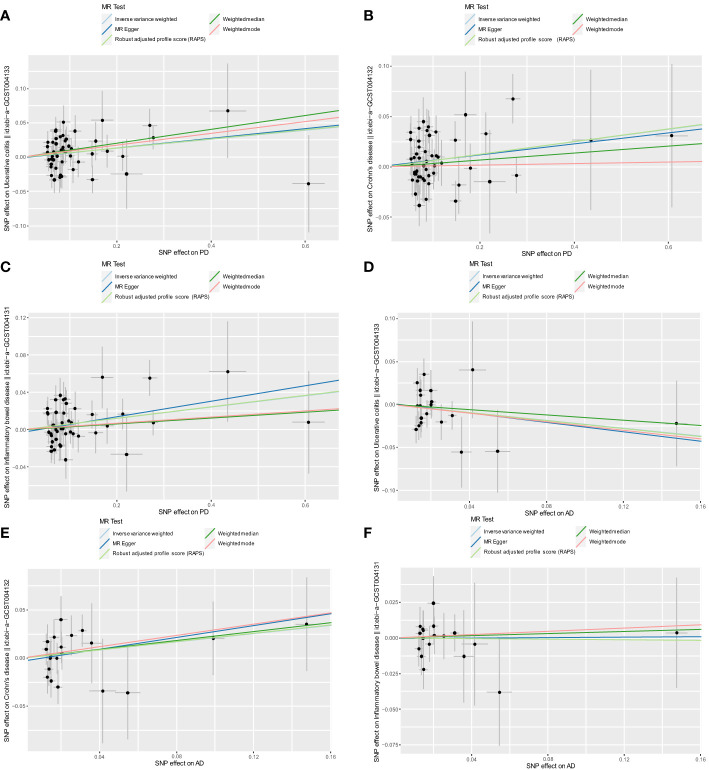
Scatter plots for MR analyses of the causal effect of PD and AD on IBD. **(A)** PD-UC. **(B)** PD-CD. **(C)** PD-IBD. **(D)** AD-UC. **(E)** AD-CD. **(F)** AD-IBD. Analyses were conducted using the conventional IVW, WM, MR-Egger, RAPS, and weighted mode methods. The slope of each line corresponds to the estimated MR effect per method. PD, Parkinson’s disease; AD, Alzheimer’s disease; IBD, inflammatory bowel disease; MR, Mendelian randomization; IVW, inverse variance weighted; UC, ulcerative colitis; CD, Crohn’s disease; RAPS, Robust Adjusted Profile Score.

After excluding outlier SNPs through the MR-PRESSO global test, we selected 50, 51, and 47 SNPs to explore the causal effects of genetically predicted PD on UC, CD, and IBD (**Supplementary Tables S3–S5**), respectively. The causal effects of genetically predicted PD on IBD (UC and CD) were inconsistent among the five MR methods. Specifically, the results of the IVW, WM, and RAPS methods showed that genetically predicted PD was significantly associated with an increased risk of UC (odds ratio [OR]_IVW_=1.068, OR_WM_=1.107, OR_RAPS_=1.069, all *P*<0.05). However, using MR-Egger and weighted mode methods, we found no significant association between genetically predicted PD and UC. Additionally, we found a significant association between genetically predicted PD and CD (OR_IVW_=1.064, OR_RAPS_=1.065) and IBD (OR_IVW_=1.062, OR_RAPS_=1.063) using IVW and RAPS methods. However, these significant associations were not replicated by the other three methods. The scatter plots displayed the single SNP effect and the combined effects of each MR method (**Figures 2A**–**C**). Forest plots and funnel plots of the causal effect of genetically predicted PD on IBD (UC and CD) are displayed in **Supplementary Figures S1–S3**, **S7–S9**. More details of the MR analysis of the causal effects of genetically predicted PD on IBD (UC and CD) are shown in **Table 1**.

**Table 1:** **Mendelian randomization estimates for causal effects of PD on IBD**.

Effect	MR Methods	β	OR	95%CI	P-Value	Q-statistic	*P*-Value
PD on UC	MR Egger	0.070	1.073	0.964-1.193	0.204	54.956	0.228
	Weighted median	0.101	1.107	1.021-1.199	0.013		
	Inverse variance weighted	0.066	1.068	1.013-1.125	0.014	54.965	0.259
	RAPS	0.067	1.069	1.017-1.124	0.009		
	Weighted mode	0.086	1.090	0.995-1.194	0.069		
PD on CD	MR Egger	0.055	1.057	0.945-1.182	0.340	60.698	0.122
	Weighted median	0.035	1.035	0.956-1.122	0.396		
	Inverse variance weighted	0.062	1.064	1.007-1.124	0.026	60.722	0.142
	RAPS	0.063	1.065	1.013-1.119	0.014		
	Weighted mode	0.008	1.008	0.900-1.129	0.892		
PD on IBD	MR Egger	0.083	1.086	0.995-1.186	0.072	55.318	0.139
	Weighted median	0.031	1.031	0.967-1.100	0.350		
	Inverse variance weighted	0.061	1.062	1.017-1.110	0.007	55.724	0.154
	RAPS	0.061	1.063	1.021-1.107	0.003		
	Weighted mode	0.033	1.034	0.962-1.111	0.370		

PD, Parkinson’s disease; IBD, inflammatory bowel disease; MR, Mendelian randomization; OR, odds ratio; CI, confidence interval; UC, ulcerative colitis; CD, Crohn’s disease; RAPS, Robust Adjusted Profile Score.

### Causal effects of AD on IBD

After excluding outlier SNPs through the MR-PRESSO global test, 20, 21, and 18 SNPs were selected to explore the causal effects of genetically predicted AD on UC, CD, and IBD (**Supplementary Tables S6–S8**), respectively. The scatter plots display the single SNP effect and combined effects of each MR method, as shown in **Figures 2D**–**F**. The results of the five MR methods showed no significant association between genetically predicted AD and IBD (**Table 2**). Forest plots and funnel plots of the causal effect of genetically predicted AD on IBD (UC and CD) are displayed in **Supplementary Figures S4–S6**, **S10**–**S12**. Based on these results, we concluded that there was no evidence of a causal association between genetically predicted AD and IBD.

**Table 2:** Mendelian randomization estimates for causal effects of AD on IBD.

Effect	MR Methods	β	OR	95%CI	P-Value	Q-statistic	*P*-Value
AD on UC	MR Egger	-0.274	0.761	0.383-1.509	0.444	19.927	0.337
	Weighted median	-0.152	0.859	0.514-1.436	0.561		
	Inverse variance weighted	-0.232	0.793	0.544-1.155	0.227	19.950	0.398
	RAPS	-0.235	0.790	0.545-1.147	0.216		
	Weighted mode	-0.251	0.778	0.457-1.324	0.366		
AD on CD	MR Egger	0.308	1.361	0.800-2.315	0.270	16.813	0.603
	Weighted median	0.231	1.260	0.804-1.975	0.314		
	Inverse variance weighted	0.213	1.237	0.890-1.720	0.205	17.014	0.652
	RAPS	0.215	1.240	0.888-1.730	0.207		
	Weighted mode	0.294	1.342	0.833-2.161	0.240		
AD on IBD	MR Egger	0.008	1.008	0.606 1.678	0.975	8.332	0.938
	Weighted median	0.038	1.039	0.694 1.556	0.853		
	Inverse variance weighted	-0.010	0.990	0.736 1.331	0.945	8.340	0.959
	RAPS	-0.010	0.990	0.733 1.337	0.946		
	Weighted mode	0.058	1.060	0.688 1.632	0.794		

AD, Alzheimer’s disease; IBD, inflammatory bowel disease; MR, Mendelian randomization; OR, odds ratio; CI, confidence interval; UC, ulcerative colitis; CD, Crohn’s disease; RAPS, Robust Adjusted Profile Score.

### Sensitivity analysis

To further verify the reliability of the above results, we performed pleiotropy, heterogeneity, and sensitivity analysis. The MR-Egger intercept and MR-PRESSO global tests showed no horizontal pleiotropy (all *P*>0.05). Furthermore, no statistical heterogeneity was found in the heterogeneity test of MR-Egger and IVW methods. The results of the leave-one-out sensitivity analysis showed that the causal effect estimation of genetically predicted PD and AD on IBD was robust. The results of the sensitivity analysis are shown in **Supplementary Table S9** and **Supplementary Figures S13–S18**.

## Discussion

The IVW and RAPS methods supported the notion that there were causal associations of genetically predicted PD with UC, CD, and IBD. This MR provided estimates of the risk factor effect over a lifetime (44), which could be interpreted as the effect on IBD after the onset of AD and PD at a specific age. However, it should be noted that our finding was contrary to the findings of previous epidemiological studies. Camacho-Soto et al. performed a case-control study based on comprehensive American Medicare data from 2004 to 2009 (24), which found that PD was associated with a lower risk of IBD. However, owing to the cross-sectional design of the above study, the actual sequence and time of onset, complete evolution, development, and subsequent treatment history of PD and IBD were not included in the aforementioned data. In addition, gastrointestinal symptoms, including dysphagia, delayed gastric emptying, and constipation, are common in patients with PD, which means that the typical IBD symptoms (e.g., diarrhea) could be partially hidden, making the diagnosis challenging (9). These factors may be important reasons for the negative association observed between the risk of IBD and PD in the above study.

Based on previous studies, the relationship between PD and an increased risk of IBD may involve the following aspects. First, they have a common genetic susceptibility. Leucine-rich repeat kinase 2 (*LRRK2*) is one of the most common pathogenic genes involved in PD. The *M2397T* variant affects LRRK2 protein levels and is associated with IBD (45). The variant *N2081D* has shared effects with increased risks of PD and CD, whereas *R1398H* and *N551K* were related to reduced risks of PD and CD (46, 47). The two susceptibility loci (FCGR2A and NOD2) identified in the largest GWAS meta-analysis of PD are also closely related to IBD pathogenesis (9, 31). Furthermore, by analyzing the GWAS results of patients with PD, UC, and CD, Witoelar et al. found that there were genetic overlaps between PD, UC (*BTNL2*, *HLA*, *GUCY1A3*, and *TRIM10*), and CD (*LRRK2*, *MROH3P*, *MAPT*, *RSPH6A*, *HLA*, *SYMPK*, and *CCNY*) (48), indicating that these three have partially common genetic pathways. Second, there are tripartite communications among the brain, gut, and microbiome, in which the imbalance of the gut microbiota plays a key role. After central dopaminergic nigrostriatal neurodegeneration in rats induced by injection of 6-hydroxydopamine, bowel inflammation and oxidative stress increased, accompanied by increased pro-inflammatory cytokine levels (15). Thereafter, a previous study observed that the colon tissue of the rat model of PD displayed significant changes, including inflammatory infiltration, intestinal barrier injury (reduced claudin-1 and transmembrane 16A/anoctamin 1 expressed by epithelial cells and a compensatory increase of mucin and S100-positive glial cells), and enhanced transmural deposition of collagen fibers (49).

PD may lead to an intestinal inflammatory response, mucosal barrier damage, and immune activation. Prior meta-analysis of the gut microbiome data set of patients with PD indicated alterations of high abundance of genera *Akkermansia*, *Lactobacillus*, and *Bifidobacterium*, as well as a lower abundance of important short-chain fatty acid producers, including *Roseburia*, *Blautia*, and *Anaerostipes* (*Lachnospiraceae* family) and *Faecalibacterium* (*Ruminococcaceae* family) (50). These imbalances may lead to immune reactions mediated by T cells (51), an intestinal pro-inflammatory state (52), and changes in gut mucosal permeability, which then develop into IBD. Third, the interaction between the central nervous system and enteric glial cells in the enteric nervous system and the neuroimmune crosstalk seem to partially explain the association between PD and IBD. Studies have shown increased expression of enteric glial markers (glial fibrillary acidic protein and SRY-related HMG-box 10) in patients with PD (53, 54). This reactive enteric gliosis can release pro-inflammatory cytokines such as interleukin (IL)-1β and IL-6, tumor necrosis factor (TNF), glial cell-derived neurotrophic factor, and other immunomodulatory signaling molecules such as nitric oxide and S100B, all of which contribute to intestinal inflammation (55).

Additionally, the role of enteric glial cells in presenting antigens may regulate immune responses mediated by T cells (51). However, the specific effect and mechanism remain unclear. Fourth, recent research has suggested that autophagy and mitochondrial as well as lysosomal dysfunction in the pathogenesis of PD also seem to be involved in pathological links in IBD, such as a damaged intestinal epithelial barrier, endoplasmic reticulum stress response in enterocytes, and dysfunction of Paneth cells and immune cells (9, 56, 57). In sum, the underlying mechanism behind the relationship between PD and IBD is complex and worthy of further investigation.

In the present study, there was no evidence of a causal association between genetically predicted AD and IBD. Previous studies have regarded IBD as exposure to explore its association with AD, but few studies have explored the reverse association. For example, a meta-analysis showed that patients with IBD were more likely to have AD (58). To the best of our knowledge, our study is the first to explore the causal effect of AD on IBD, further deepening our understanding of the association between AD and IBD. However, there was no causal association between AD and IBD, although this finding has some possible implications in previous studies. A previous meta-analysis of cytokine in AD showed that levels of inflammatory factors such as IL-6 and TNF were significantly increased in patients with AD (59), and a number of studies suggested that inflammatory responses were one of the pathogenesis of IBD (60). In addition, a previous study found that the gut microbiome of patients with AD significantly changed compared to that of the general population, mainly including a decrease in *Firmicutes* (61). Another study suggested that dysbacteriosis or clear changes in the healthy gut microbiome may be the decisive event in the occurrence and development of IBD (62). Previous studies have also found that the most definite change in the gut microbiome of patients with IBD is the decrease in the abundance of *Firmicutes* (63–65). To a certain extent, these studies support the association of AD with IBD. However, owing to the lack of direct evidence, existing studies could not infer a causal association of AD on IBD. Based on the level of evidence and study design, our MR analysis strongly supports that a causal effect of AD on IBD does not exist in people of European descent. Further studies are needed to explore whether AD has a causal effect on IBD.

This study has several limitations. First, in our MR analysis, PD, AD, and IBD came from two samples. It should be noted that the GWAS data of PD, AD, and IBD that were selected were all the results of meta-analyses, which had been adjusted for age and sex, and all of them contain data from the UK Biobank, meaning there may be a sample overlap. Although all the IVs we selected were strong, it is undeniable that sample overlap may have led to bias. Second, our MR study showed that genetically predicted PD had a causal effect on IBD, but the result of the MR analysis was only genetic evidence. This possible causal association and its related mechanisms must be further explored and verified in animal experiments or population-based observational studies. Considering that evidence from observational studies on the associations of PD and AD with IBD was limited, the causal effects of PD and AD on IBD should be supported by cohort studies. For example, cohorts of PD/AD and non-PD/AD patients (without IBD) could be constructed to observe the difference in the incidence of IBD between the groups during follow-up, which would help to explore the longitudinal association between PD/AD at baseline and IBD at follow-up. Third, the genetic tools and GWAS population selected in this study were based on Europeans, which means that whether the results can be generalized to other ethnicities remains to be verified.

Nevertheless, our study had several strengths. First, we selected large-scale GWAS data for the MR analysis, which reduced the bias caused by population stratification. Second, we selected independent and strong genetic variants as IVs to reduce the impact of LD and weak instruments bias. Third, we used various MR methods for the first time, which provided strong support for exploring the causal effects of genetically predicted PD and AD on IBD. Neurodegenerative diseases, e.g., PD and AD, mostly occur in the elderly population, whereas IBD mostly occurs in middle-aged and young individuals. Therefore, traditional observational studies need to exclude IBD from the baseline PD/AD population for follow-up and explore the potential causal relationship between baseline PD/AD and IBD at follow-up. However, it should be noted that if the aforementioned design is followed, it is often time-consuming, expensive, and labor intensive. Our MR study used genetic variants as the IV to avoid the aforementioned problems without considering the sequence of onset time.

## Conclusion

Our MR study corroborated a causal association between genetically predicted PD and IBD but did not support a causal effect of genetically predicted AD on IBD. More animal experiments or population-based observational studies are required to clarify the underlying mechanisms of PD and IBD.

## Data availability statement

Publicly available datasets were analyzed in this study. This data can be found here: Website of the ieu open gwas project (https://gwas.mrcieu.ac.uk/), the GWAS summary data of Alzheimer’s disease (https://ctg.cncr.nl/software/summary_statistics) and publication of the International Parkinson’s Disease Genomics Consortium (https://doi.org/10.1016/S1474-4422(19)30320-5).

## Author contributions

GC, SL and XZ contributed to the study design. GC and SL analyzed the data and drafted the manuscript. YY, QH, YC, and ZS were involved in compiling the data and gave comments on the draft. HY and XZ revised the draft. All authors read and approved the final manuscript.

## Acknowledgments

Thanks to International IBD Genetics Consortium, International Parkinson’s Disease Genomics Consortium, the International Genomics of Alzheimer’s Project, Alzheimer’s Disease Sequencing Project, Alzheimer’s Disease Working Group of the Psychiatric Genomics Consortium, and UK Biobank for the GWAS data. Thank Dr. Feixiang Zhou for his guidance and help in revising our manuscript.

## Conflict of interest

The authors declare that the research was conducted in the absence of any commercial or financial relationships that could be construed as a potential conflict of interest.

## Publisher’s note

All claims expressed in this article are solely those of the authors and do not necessarily represent those of their affiliated organizations, or those of the publisher, the editors and the reviewers. Any product that may be evaluated in this article, or claim that may be made by its manufacturer, is not guaranteed or endorsed by the publisher.
