# Supplementary material for: Are neurodegenerative diseases associated with an increased risk of inflammatory bowel disease? A two-sample Mendelian randomization study
Source: Front Immunol. 2022 Sep 8;13:956005. doi: 10.3389/fimmu.2022.956005 (PMC9493012; doi:10.3389/fimmu.2022.956005)
Supplement: Supplementary file 1 [file DataSheet_1.pdf]

Table S1 Genetic variants used as instrumental variables for PD

SNP	A1	A2	EAF	*β*	S.E.	*P*-value	F-statistic
rs10513789	T	G	0.8112	0.1485	0.0121	1.22E-34	150.620
rs10748818	A	G	0.8514	-0.0790	0.0130	1.05E-09	36.929
rs10797576	T	C	0.1403	0.1114	0.0133	6.84E-17	70.156
rs10847864	T	G	0.3640	0.1478	0.0115	1.47E-37	165.178
rs11150601	A	G	0.6442	0.0907	0.0099	5.12E-20	83.935
rs11158026	T	C	0.3245	-0.0842	0.0102	1.66E-16	68.143
rs11578699	T	C	0.1949	-0.0704	0.0120	4.47E-09	34.418
rs117896735	A	G	0.0166	0.4354	0.0394	2.36E-28	122.119
rs11950533	A	C	0.1020	-0.0916	0.0158	7.16E-09	33.611
rs12147950	T	C	0.4376	-0.0529	0.0096	3.54E-08	30.365
rs12283611	A	C	0.4148	-0.0645	0.0102	2.61E-10	39.987
rs12456492	A	G	0.6816	-0.0983	0.0099	3.80E-23	98.591
rs12497850	T	G	0.6476	0.0636	0.0099	1.36E-10	41.271
rs12528068	T	C	0.2844	0.0657	0.0103	1.63E-10	40.687
rs12600861	A	C	0.6484	-0.0565	0.0099	1.01E-08	32.571
rs1293298	A	C	0.7444	0.0930	0.0114	3.99E-16	66.551
rs13117519	T	C	0.1744	0.0875	0.0123	9.82E-13	50.606
rs13294100	T	G	0.3422	-0.0859	0.0100	8.72E-18	73.788
rs1474055	T	C	0.1312	0.1796	0.0137	2.54E-39	171.858
rs1867598	A	G	0.9019	-0.1554	0.0156	2.52E-23	99.232
rs1941685	T	G	0.4983	0.0531	0.0094	1.69E-08	31.910
rs199351	A	C	0.5939	0.1016	0.0096	5.25E-26	112.007
rs2086641	T	C	0.7225	-0.0605	0.0107	1.81E-08	31.970
rs2248244	A	G	0.2828	0.0714	0.0107	2.74E-11	44.527
rs2251086	T	C	0.1417	-0.1186	0.0137	6.08E-18	74.942
rs2280104	T	C	0.3604	0.0556	0.0098	1.16E-08	32.188
rs34311866	T	C	0.8065	-0.2126	0.0120	9.98E-70	313.880
rs356182	A	G	0.6278	-0.2774	0.0105	3.89E-154	697.965
rs35749011	A	G	0.0169	0.6068	0.0342	1.72E-70	314.803
rs3742785	A	C	0.7866	0.0707	0.0118	1.92E-09	35.898
rs3802920	T	G	0.2054	0.1073	0.0117	6.26E-20	84.106
rs4653767	T	C	0.7196	0.0833	0.0104	1.38E-15	64.154
rs4698412	A	G	0.5529	0.1035	0.0094	2.06E-28	121.234
rs4771268	T	C	0.2295	0.0675	0.0112	1.45E-09	36.322
rs55818311	T	C	0.6937	-0.0696	0.0111	4.18E-10	39.316
rs55961674	T	C	0.1722	0.0861	0.0126	9.98E-12	46.694
rs57891859	A	G	0.7185	0.0807	0.0107	4.55E-14	56.883
rs61169879	T	C	0.1641	0.0820	0.0134	9.28E-10	37.447
rs62053943	T	C	0.1552	-0.2700	0.0155	3.58E-68	303.434
rs6476434	T	C	0.7336	-0.0615	0.0106	6.58E-09	33.662
rs6808178	T	C	0.3794	0.0658	0.0096	8.09E-12	46.980
rs6854006	T	C	0.3632	-0.0912	0.0097	5.82E-21	88.399

rs7134559	T	C	0.4040	-0.0539	0.0098	3.96E-08	30.250
rs73038319	A	C	0.9592	-0.1693	0.0235	5.94E-13	51.901
rs75859381	T	C	0.9673	-0.2207	0.0341	1.04E-10	41.889
rs76904798	T	C	0.1444	0.1439	0.0130	1.52E-28	122.528
rs77351827	T	C	0.1275	0.0802	0.0139	8.87E-09	33.290
rs7938782	A	G	0.8776	0.0870	0.0145	2.12E-09	36.000
rs823118	T	C	0.5660	0.1066	0.0094	1.11E-29	128.605
rs850738	A	G	0.6056	-0.0710	0.0105	1.29E-11	45.723
rs896435	T	C	0.6892	0.0735	0.0101	3.41E-13	52.958
rs9568188	T	C	0.7397	0.0617	0.0108	1.15E-08	32.638
rs979812	T	G	0.4421	0.0610	0.0093	6.19E-11	43.022
rs997368	A	G	0.8049	0.0714	0.0119	1.84E-09	36.000

TableS2 Genetic variants used as instrumental variables for AD

SNP	A1	A2	EAF	*β*	S.E.	*P*-value	F-statistic
rs11218343	C	T	0.0441	-0.0359	0.0053	8.12E-12	46.737
rs11257238	C	T	0.3610	0.0129	0.0023	1.04E-08	32.761
rs113260531	A	G	0.1261	0.0200	0.0033	7.91E-10	37.783
rs118170342	C	T	0.0374	0.1475	0.0057	7.93E-148	670.458
rs12590654	A	G	0.3365	-0.0148	0.0023	1.32E-10	41.275
rs1859788	A	G	0.3243	-0.0184	0.0023	1.8E-15	63.267
rs204473	A	G	0.0244	-0.0417	0.0070	2.58E-09	35.476
rs2081545	A	C	0.3821	-0.0179	0.0022	1.11E-15	64.230
rs28394864	A	G	0.4533	0.0123	0.0022	1.68E-08	31.828
rs28399657	G	A	0.0315	-0.0546	0.0066	9.82E-17	69.004
rs41290120	A	G	0.0362	-0.0991	0.0058	7.14E-66	293.866
rs4236673	A	G	0.3778	-0.0202	0.0022	1.48E-19	81.834
rs442495	C	T	0.3543	-0.0137	0.0023	1.22E-09	36.935
rs4575098	A	G	0.2280	0.0164	0.0026	1.9E-10	40.570
rs4663105	C	A	0.4113	0.0311	0.0022	1.45E-44	196.149
rs59735493	A	G	0.2985	-0.0130	0.0024	3.73E-08	30.286
rs6014724	G	A	0.0950	-0.0229	0.0037	5.38E-10	38.534
rs6448453	A	G	0.2625	0.0147	0.0025	1.98E-09	35.997
rs679515	T	C	0.1715	0.0254	0.0029	6.83E-19	78.811
rs755951	C	A	0.4130	0.0150	0.0022	1.13E-11	46.083
rs7810606	T	C	0.4854	-0.0145	0.0022	2.89E-11	44.249
rs867611	G	A	0.3174	-0.0204	0.0023	1.48E-18	77.282
rs9381563	C	T	0.3557	0.0145	0.0023	1.99E-10	40.472

TableS3 Summary of genetic variants used to estimate the effects of PD on UC in MR analysis

			Association with PD			Association with UC		
SNP	A1/A2	EAF	*β*	S.E	*P*-value	*β*	S.E	*P*-value
rs10513789	T/G	0.8112	0.1485	0.0121	1.22E-34	-0.0326	0.0198	0.0993
rs10748818	A/G	0.8514	-0.0790	0.0130	1.05E-09	0.0294	0.0223	0.1887
rs10797576	T/C	0.1403	0.1114	0.0133	6.84E-17	0.0380	0.0232	0.1018

rs10847864	T/G	0.3640	0.1478	0.0115	1.47E-37	0.0048	0.0192	0.8042
rs11150601	A/G	0.6442	0.0907	0.0099	5.12E-20	0.0239	0.0162	0.1411
rs11158026	T/C	0.3245	-0.0842	0.0102	1.66E-16	0.0067	0.0168	0.6889
rs11578699	T/C	0.1949	-0.0704	0.0120	4.47E-09	-0.0379	0.0199	0.0567
rs117896735	A/G	0.0166	0.4354	0.0394	2.36E-28	0.0674	0.0686	0.3263
rs12147950	T/C	0.4376	-0.0529	0.0096	3.54E-08	-0.0067	0.0159	0.6729
rs12283611	A/C	0.4148	-0.0645	0.0102	2.61E-10	-0.0216	0.0162	0.1821
rs12456492	A/G	0.6816	-0.0983	0.0099	3.8E-23	-0.0161	0.0169	0.3408
rs12497850	T/G	0.6476	0.0636	0.0099	1.36E-10	0.0010	0.0164	0.9535
rs12528068	T/C	0.2844	0.0657	0.0103	1.63E-10	-0.0040	0.0175	0.8192
rs1293298	A/C	0.7444	0.0930	0.0114	3.99E-16	0.0264	0.0185	0.1539
rs13117519	T/C	0.1744	0.0875	0.0123	9.82E-13	0.0088	0.0214	0.6820
rs13294100	T/G	0.3422	-0.0859	0.0100	8.72E-18	-0.0140	0.0166	0.3987
rs1474055	T/C	0.1312	0.1796	0.0137	2.54E-39	0.0087	0.0242	0.7197
rs1867598	A/G	0.9019	-0.1554	0.0156	2.52E-23	-0.0234	0.0281	0.4051
rs1941685	T/G	0.4983	0.0531	0.0094	1.69E-08	0.0153	0.0158	0.3339
rs199351	A/C	0.5939	0.1016	0.0096	5.25E-26	0.0018	0.0160	0.9082
rs2086641	T/C	0.7225	-0.0605	0.0107	1.81E-08	0.0140	0.0180	0.4361
rs2248244	A/G	0.2828	0.0714	0.0107	2.74E-11	0.0268	0.0176	0.1280
rs2251086	T/C	0.1417	-0.1186	0.0137	6.08E-18	0.0070	0.0225	0.7549
rs2280104	T/C	0.3604	0.0556	0.0098	1.16E-08	-0.0040	0.0165	0.8058
rs34311866	T/C	0.8065	-0.2126	0.0120	9.98E-70	-0.0012	0.0214	0.9555
rs356182	A/G	0.6278	-0.2774	0.0105	3.89E-154	-0.0285	0.0171	0.0958
rs35749011	A/G	0.0169	0.6068	0.0342	1.72E-70	-0.0387	0.0708	0.5842
rs3742785	A/C	0.7866	0.0707	0.0118	1.92E-09	0.0396	0.0191	0.0380
rs3802920	T/G	0.2054	0.1073	0.0117	6.26E-20	-0.0181	0.0200	0.3648
rs4653767	T/C	0.7196	0.0833	0.0104	1.38E-15	-0.0284	0.0175	0.1041
rs4698412	A/G	0.5529	0.1035	0.0094	2.06E-28	0.0122	0.0159	0.4422
rs4771268	T/C	0.2295	0.0675	0.0112	1.45E-09	-0.0331	0.0191	0.0835
rs55818311	T/C	0.6937	-0.0696	0.0111	4.18E-10	-0.0052	0.0189	0.7851
rs55961674	T/C	0.1722	0.0861	0.0126	9.98E-12	0.0315	0.0218	0.1476
rs57891859	A/G	0.7185	0.0807	0.0107	4.55E-14	-0.0266	0.0180	0.1409
rs61169879	T/C	0.1641	0.0820	0.0134	9.28E-10	0.0102	0.0219	0.6407
rs62053943	T/C	0.1552	-0.2700	0.0155	3.58E-68	-0.0462	0.0245	0.0593
rs6476434	T/C	0.7336	-0.0615	0.0106	6.58E-09	0.0030	0.0177	0.8655
rs6808178	T/C	0.3794	0.0658	0.0096	8.09E-12	0.0074	0.0163	0.6482
rs6854006	T/C	0.3632	-0.0912	0.0097	5.82E-21	-0.0071	0.0163	0.6639
rs7134559	T/C	0.4040	-0.0539	0.0098	3.96E-08	-0.0216	0.0163	0.1855
rs73038319	A/C	0.9592	-0.1693	0.0235	5.94E-13	-0.0538	0.0428	0.2089
rs75859381	T/C	0.9673	-0.2207	0.0341	1.04E-10	0.0245	0.0503	0.6254
rs77351827	T/C	0.1275	0.0802	0.0139	8.87E-09	0.0295	0.0240	0.2203
rs7938782	A/G	0.8776	0.0870	0.0145	2.12E-09	0.0510	0.0243	0.0361
rs850738	A/G	0.6056	-0.0710	0.0105	1.29E-11	0.0161	0.0166	0.3327
rs896435	T/C	0.6892	0.0735	0.0101	3.41E-13	0.0014	0.0169	0.9335

rs9568188	T/C	0.7397	0.0617	0.0108	1.15E-08	-0.0268	0.0180	0.1378
rs979812	T/G	0.4421	0.0610	0.0093	6.19E-11	-0.0103	0.0159	0.5151
rs997368	A/G	0.8049	0.0714	0.0119	1.84E-09	0.0194	0.0205	0.3439

TableS4 Summary of genetic variants used to estimate the effects of PD on CD in MR analysis

			Association with PD			Association with CD		
SNP	A1/A2	EAF	β	S.E	*P*-value	β	S.E	*P*-value
rs10513789	T/G	0.8112	0.1485	0.0121	1.22E-34	-0.0339	0.0201	0.0919
rs10748818	A/G	0.8514	-0.0790	0.0130	1.05E-09	0.0118	0.0226	0.6026
rs10797576	T/C	0.1403	0.1114	0.0133	6.84E-17	0.0098	0.0237	0.6780
rs10847864	T/G	0.3640	0.1478	0.0115	1.47E-37	0.0266	0.0190	0.1617
rs11150601	A/G	0.6442	0.0907	0.0099	5.12E-20	0.0398	0.0164	0.0156
rs11158026	T/C	0.3245	-0.0842	0.0102	1.66E-16	-0.0152	0.0171	0.3749
rs11578699	T/C	0.1949	-0.0704	0.0120	4.47E-09	-0.0257	0.0200	0.1983
rs117896735	A/G	0.0166	0.4354	0.0394	2.36E-28	0.0266	0.0697	0.7024
rs11950533	A/C	0.1020	-0.0916	0.0158	7.16E-09	0.0088	0.0263	0.7390
rs12147950	T/C	0.4376	-0.0529	0.0096	3.54E-08	-0.0272	0.0161	0.0925
rs12283611	A/C	0.4148	-0.0645	0.0102	2.61E-10	0.0073	0.0164	0.6563
rs12456492	A/G	0.6816	-0.0983	0.0099	3.8E-23	-0.0111	0.0171	0.5160
rs12497850	T/G	0.6476	0.0636	0.0099	1.36E-10	-0.0270	0.0167	0.1055
rs12528068	T/C	0.2844	0.0657	0.0103	1.63E-10	0.0130	0.0178	0.4642
rs12600861	A/C	0.6484	-0.0565	0.0099	1.01E-08	0.0130	0.0169	0.4408
rs1293298	A/C	0.7444	0.0930	0.0114	3.99E-16	0.0313	0.0186	0.0935
rs13117519	T/C	0.1744	0.0875	0.0123	9.82E-13	0.0362	0.0216	0.0938
rs13294100	T/G	0.3422	-0.0859	0.0100	8.72E-18	0.0025	0.0169	0.8804
rs1474055	T/C	0.1312	0.1796	0.0137	2.54E-39	-0.0014	0.0246	0.9544
rs1867598	A/G	0.9019	-0.1554	0.0156	2.52E-23	0.0180	0.0285	0.5288
rs1941685	T/G	0.4983	0.0531	0.0094	1.69E-08	0.0344	0.0160	0.0320
rs199351	A/C	0.5939	0.1016	0.0096	5.25E-26	-0.0062	0.0162	0.6999
rs2086641	T/C	0.7225	-0.0605	0.0107	1.81E-08	-0.0092	0.0181	0.6109
rs2248244	A/G	0.2828	0.0714	0.0107	2.74E-11	0.0088	0.0179	0.6244
rs2251086	T/C	0.1417	-0.1186	0.0137	6.08E-18	-0.0038	0.0228	0.8676
rs2280104	T/C	0.3604	0.0556	0.0098	1.16E-08	0.0034	0.0167	0.8380
rs34311866	T/C	0.8065	-0.2126	0.0120	9.98E-70	-0.0329	0.0212	0.1202
rs356182	A/G	0.6278	-0.2774	0.0105	3.89E-154	0.0085	0.0174	0.6251
rs35749011	A/G	0.0169	0.6068	0.0342	1.72E-70	0.0309	0.0712	0.6644
rs3742785	A/C	0.7866	0.0707	0.0118	1.92E-09	0.0384	0.0194	0.0477
rs3802920	T/G	0.2054	0.1073	0.0117	6.26E-20	0.0110	0.0200	0.5843
rs4698412	A/G	0.5529	0.1035	0.0094	2.06E-28	0.0011	0.0160	0.9477
rs4771268	T/C	0.2295	0.0675	0.0112	1.45E-09	-0.0139	0.0193	0.4702
rs55818311	T/C	0.6937	-0.0696	0.0111	4.18E-10	0.0098	0.0186	0.6005
rs55961674	T/C	0.1722	0.0861	0.0126	9.98E-12	-0.0321	0.0220	0.1449
rs57891859	A/G	0.7185	0.0807	0.0107	4.55E-14	0.0011	0.0182	0.9527
rs61169879	T/C	0.1641	0.0820	0.0134	9.28E-10	-0.0135	0.0224	0.5462
rs62053943	T/C	0.1552	-0.2700	0.0155	3.58E-68	-0.0676	0.0247	0.0063

rs6476434	T/C	0.7336	-0.0615	0.0106	6.58E-09	0.0070	0.0180	0.6986
rs6808178	T/C	0.3794	0.0658	0.0096	8.09E-12	-0.0042	0.0165	0.7985
rs6854006	T/C	0.3632	-0.0912	0.0097	5.82E-21	0.0167	0.0165	0.3139
rs7134559	T/C	0.4040	-0.0539	0.0098	3.96E-08	-0.0077	0.0166	0.6433
rs73038319	A/C	0.9592	-0.1693	0.0235	5.94E-13	-0.0518	0.0427	0.2247
rs75859381	T/C	0.9673	-0.2207	0.0341	1.04E-10	0.0147	0.0514	0.7756
rs77351827	T/C	0.1275	0.0802	0.0139	8.87E-09	0.0449	0.0241	0.0628
rs7938782	A/G	0.8776	0.0870	0.0145	2.12E-09	0.0057	0.0241	0.8123
rs823118	T/C	0.5660	0.1066	0.0094	1.11E-29	0.0347	0.0162	0.0319
rs850738	A/G	0.6056	-0.0710	0.0105	1.29E-11	-0.0196	0.0168	0.2441
rs896435	T/C	0.6892	0.0735	0.0101	3.41E-13	-0.0099	0.0171	0.5624
rs9568188	T/C	0.7397	0.0617	0.0108	1.15E-08	-0.0053	0.0183	0.7720
rs997368	A/G	0.8049	0.0714	0.0119	1.84E-09	-0.0384	0.0204	0.0592

TableS5 Summary of genetic variants used to estimate the effects of PD on IBD in MR analysis

			Association with PD			Association with IBD		
SNP	A1/A2	EAF	β	S.E	*P*-value	β	S.E	*P*-value
rs10748818	A/G	0.8514	-0.0790	0.0130	1.05E-09	0.0165	0.0175	0.3456
rs10797576	T/C	0.1403	0.1114	0.0133	6.84E-17	0.0226	0.0183	0.2160
rs10847864	T/G	0.3640	0.1478	0.0115	1.47E-37	0.0162	0.0149	0.2753
rs11150601	A/G	0.6442	0.0907	0.0099	5.12E-20	0.0329	0.0128	0.0100
rs11158026	T/C	0.3245	-0.0842	0.0102	1.66E-16	-0.0072	0.0132	0.5865
rs11578699	T/C	0.1949	-0.0704	0.0120	4.47E-09	-0.0322	0.0156	0.0390
rs117896735	A/G	0.0166	0.4354	0.0394	2.36E-28	0.0622	0.0538	0.2482
rs11950533	A/C	0.1020	-0.0916	0.0158	7.16E-09	0.0324	0.0202	0.1089
rs12147950	T/C	0.4376	-0.0529	0.0096	3.54E-08	-0.0182	0.0125	0.1453
rs12283611	A/C	0.4148	-0.0645	0.0102	2.61E-10	-0.0043	0.0127	0.7341
rs12456492	A/G	0.6816	-0.0983	0.0099	3.8E-23	-0.0092	0.0133	0.4903
rs12497850	T/G	0.6476	0.0636	0.0099	1.36E-10	-0.0132	0.0129	0.3050
rs12528068	T/C	0.2844	0.0657	0.0103	1.63E-10	0.0049	0.0138	0.7229
rs1293298	A/C	0.7444	0.0930	0.0114	3.99E-16	0.0282	0.0145	0.0519
rs13117519	T/C	0.1744	0.0875	0.0123	9.82E-13	0.0176	0.0168	0.2941
rs13294100	T/G	0.3422	-0.0859	0.0100	8.72E-18	-0.0019	0.0131	0.8848
rs1474055	T/C	0.1312	0.1796	0.0137	2.54E-39	0.0039	0.0190	0.8373
rs1867598	A/G	0.9019	-0.1554	0.0156	2.52E-23	0.0035	0.0220	0.8733
rs1941685	T/G	0.4983	0.0531	0.0094	1.69E-08	0.0226	0.0124	0.0691
rs199351	A/C	0.5939	0.1016	0.0096	5.25E-26	-0.0026	0.0125	0.8375
rs2086641	T/C	0.7225	-0.0605	0.0107	1.81E-08	0.0047	0.0141	0.7386
rs2248244	A/G	0.2828	0.0714	0.0107	2.74E-11	0.0184	0.0139	0.1852
rs2251086	T/C	0.1417	-0.1186	0.0137	6.08E-18	0.0070	0.0177	0.6922
rs2280104	T/C	0.3604	0.0556	0.0098	1.16E-08	-0.0028	0.0129	0.8313
rs34311866	T/C	0.8065	-0.2126	0.0120	9.98E-70	-0.0167	0.0166	0.3145
rs356182	A/G	0.6278	-0.2774	0.0105	3.89E-154	-0.0074	0.0135	0.5822
rs35749011	A/G	0.0169	0.6068	0.0342	1.72E-70	0.0079	0.0552	0.8866
rs3802920	T/G	0.2054	0.1073	0.0117	6.26E-20	-0.0045	0.0156	0.7716

rs4698412	A/G	0.5529	0.1035	0.0094	2.06E-28	0.0074	0.0124	0.5503
rs4771268	T/C	0.2295	0.0675	0.0112	1.45E-09	-0.0218	0.0150	0.1463
rs55818311	T/C	0.6937	-0.0696	0.0111	4.18E-10	-0.0026	0.0147	0.8599
rs55961674	T/C	0.1722	0.0861	0.0126	9.98E-12	0.0008	0.0171	0.9645
rs57891859	A/G	0.7185	0.0807	0.0107	4.55E-14	-0.0176	0.0142	0.2126
rs61169879	T/C	0.1641	0.0820	0.0134	9.28E-10	0.0010	0.0172	0.9544
rs62053943	T/C	0.1552	-0.2700	0.0155	3.58E-68	-0.0554	0.0192	0.0039
rs6476434	T/C	0.7336	-0.0615	0.0106	6.58E-09	0.0064	0.0139	0.6465
rs6854006	T/C	0.3632	-0.0912	0.0097	5.82E-21	0.0046	0.0128	0.7171
rs7134559	T/C	0.4040	-0.0539	0.0098	3.96E-08	-0.0170	0.0128	0.1832
rs73038319	A/C	0.9592	-0.1693	0.0235	5.94E-13	-0.0561	0.0331	0.0903
rs75859381	T/C	0.9673	-0.2207	0.0341	1.04E-10	0.0267	0.0397	0.5012
rs77351827	T/C	0.1275	0.0802	0.0139	8.87E-09	0.0367	0.0187	0.0502
rs7938782	A/G	0.8776	0.0870	0.0145	2.12E-09	0.0322	0.0189	0.0889
rs850738	A/G	0.6056	-0.0710	0.0105	1.29E-11	0.0002	0.0131	0.9890
rs896435	T/C	0.6892	0.0735	0.0101	3.41E-13	-0.0020	0.0132	0.8819
rs9568188	T/C	0.7397	0.0617	0.0108	1.15E-08	-0.0182	0.0142	0.2012
rs979812	T/G	0.4421	0.0610	0.0093	6.19E-11	-0.0234	0.0125	0.0606
rs997368	A/G	0.8049	0.0714	0.0119	1.84E-09	-0.0103	0.0159	0.5164

TableS6 Summary of genetic variants used to estimate the effects of AD on UC in MR analysis

			Association with AD			Association with UC		
SNP	A1/A2	EAF	*β*	S.E	*P*-value	*β*	S.E	*P*-value
rs11218343	C/T	0.0441	-0.0359	0.0053	8.12E-12	0.0555	0.0412	0.1777
rs11257238	C/T	0.3610	0.0129	0.0023	1.04E-08	0.0251	0.0170	0.1390
rs113260531	A/G	0.1261	0.0200	0.0033	7.91E-10	0.0162	0.0240	0.4994
rs118170342	C/T	0.0374	0.1475	0.0057	7.93E-148	-0.0223	0.0498	0.6550
rs12590654	A/G	0.3365	-0.0148	0.0023	1.32E-10	0.0214	0.0171	0.2117
rs204473	A/G	0.0244	-0.0417	0.0070	2.58E-09	-0.0403	0.0561	0.4722
rs2081545	A/C	0.3821	-0.0179	0.0022	1.11E-15	0.0110	0.0160	0.4933
rs28394864	A/G	0.4533	0.0123	0.0022	1.68E-08	-0.0291	0.0159	0.0672
rs28399657	G/A	0.0315	-0.0546	0.0066	9.82E-17	0.0546	0.0481	0.2571
rs4236673	A/G	0.3778	-0.0202	0.0022	1.48E-19	0.0003	0.0160	0.9836
rs442495	C/T	0.3543	-0.0137	0.0023	1.22E-09	0.0247	0.0168	0.1422
rs4575098	A/G	0.2280	0.0164	0.0026	1.9E-10	0.0350	0.0187	0.0613
rs4663105	C/A	0.4113	0.0311	0.0022	1.45E-44	-0.0129	0.0166	0.4356
rs59735493	A/G	0.2985	-0.0130	0.0024	3.73E-08	0.0020	0.0175	0.9102
rs6448453	A/G	0.2625	0.0147	0.0025	1.98E-09	0.0108	0.0177	0.5416
rs679515	T/C	0.1715	0.0254	0.0029	6.83E-19	-0.0205	0.0206	0.3204
rs755951	C/A	0.4130	0.0150	0.0022	1.13E-11	-0.0160	0.0162	0.3245
rs7810606	T/C	0.4854	-0.0145	0.0022	2.89E-11	0.0017	0.0165	0.9184
rs867611	G/A	0.3174	-0.0204	0.0023	1.48E-18	-0.0030	0.0167	0.8567
rs9381563	C/T	0.3557	0.0145	0.0023	1.99E-10	0.0163	0.0165	0.3250

TableS7 Summary of genetic variants used to estimate the effects of AD on CD in MR analysis

			Association with AD			Association with CD		
SNP	A1/A2	EAF	*β*	S.E	*P*-value	*β*	S.E	*P*-value
rs11218343	C/T	0.0441	-0.0359	0.0053	8.12E-12	-0.0156	0.0416	0.7080
rs11257238	C/T	0.3610	0.0129	0.0023	1.04E-08	-0.0199	0.0169	0.2377
rs113260531	A/G	0.1261	0.0200	0.0033	7.91E-10	0.0401	0.0243	0.0990
rs118170342	C/T	0.0374	0.1475	0.0057	7.93E-148	0.0352	0.0487	0.4699
rs12590654	A/G	0.3365	-0.0148	0.0023	1.32E-10	0.0237	0.0173	0.1718
rs1859788	A/G	0.3243	-0.0184	0.0023	1.8E-15	0.0301	0.0176	0.0862
rs204473	A/G	0.0244	-0.0417	0.0070	2.58E-09	0.0342	0.0544	0.5300
rs2081545	A/C	0.3821	-0.0179	0.0022	1.11E-15	0.0002	0.0162	0.9908
rs28394864	A/G	0.4533	0.0123	0.0022	1.68E-08	0.0093	0.0161	0.5622
rs28399657	G/A	0.0315	-0.0546	0.0066	9.82E-17	0.0360	0.0483	0.4569
rs41290120	A/G	0.0362	-0.0991	0.0058	7.14E-66	-0.0206	0.0382	0.5900
rs4236673	A/G	0.3778	-0.0202	0.0022	1.48E-19	-0.0114	0.0164	0.4885
rs442495	C/T	0.3543	-0.0137	0.0023	1.22E-09	0.0115	0.0172	0.5010
rs4575098	A/G	0.2280	0.0164	0.0026	1.9E-10	0.0217	0.0188	0.2499
rs4663105	C/A	0.4113	0.0311	0.0022	1.45E-44	0.0287	0.0168	0.0870
rs59735493	A/G	0.2985	-0.0130	0.0024	3.73E-08	-0.0171	0.0179	0.3407
rs6448453	A/G	0.2625	0.0147	0.0025	1.98E-09	0.0022	0.0180	0.9006
rs679515	T/C	0.1715	0.0254	0.0029	6.83E-19	0.0236	0.0209	0.2572
rs7810606	T/C	0.4854	-0.0145	0.0022	2.89E-11	0.0002	0.0166	0.9900
rs867611	G/A	0.3174	-0.0204	0.0023	1.48E-18	-0.0049	0.0170	0.7745
rs9381563	C/T	0.3557	0.0145	0.0023	1.99E-10	0.0006	0.0167	0.9717

TableS8 Summary of genetic variants used to estimate the effects of AD on IBD in MR analysis

			Association with AD			Association with IBD		
SNP	A1/A2	EAF	*β*	S.E	*P*-value	*β*	S.E	*P*-value
rs11218343	C/T	0.0441	-0.0359	0.0053	8.12E-12	0.0129	0.0324	0.6892
rs11257238	C/T	0.3610	0.0129	0.0023	1.04E-08	0.0033	0.0133	0.8058
rs113260531	A/G	0.1261	0.0200	0.0033	7.91E-10	0.0244	0.0188	0.1945
rs118170342	C/T	0.0374	0.1475	0.0057	7.93E-148	0.0036	0.0387	0.9250
rs12590654	A/G	0.3365	-0.0148	0.0023	1.32E-10	0.0222	0.0135	0.0995
rs204473	A/G	0.0244	-0.0417	0.0070	2.58E-09	0.0043	0.0432	0.9206
rs2081545	A/C	0.3821	-0.0179	0.0022	1.11E-15	0.0043	0.0126	0.7312
rs28394864	A/G	0.4533	0.0123	0.0022	1.68E-08	-0.0076	0.0125	0.5425
rs28399657	G/A	0.0315	-0.0546	0.0066	9.82E-17	0.0381	0.0375	0.3092
rs4236673	A/G	0.3778	-0.0202	0.0022	1.48E-19	-0.0083	0.0127	0.5145
rs442495	C/T	0.3543	-0.0137	0.0023	1.22E-09	0.0129	0.0132	0.3316
rs4663105	C/A	0.4113	0.0311	0.0022	1.45E-44	0.0035	0.0130	0.7891
rs59735493	A/G	0.2985	-0.0130	0.0024	3.73E-08	-0.0081	0.0138	0.5607
rs6448453	A/G	0.2625	0.0147	0.0025	1.98E-09	0.0053	0.0139	0.7021
rs679515	T/C	0.1715	0.0254	0.0029	6.83E-19	0.0014	0.0162	0.9322
rs7810606	T/C	0.4854	-0.0145	0.0022	2.89E-11	0.0003	0.0129	0.9805
rs867611	G/A	0.3174	-0.0204	0.0023	1.48E-18	-0.0018	0.0131	0.8899

rs9381563	C/T	0.3557	0.0145	0.0023	1.99E-10	0.0062	0.0130	0.6311

TableS9 Assessing pleiotropy through MR-Egger intercept and MR-PRESSO test

**Exposure**	**Outcome**	**MR-Egger intercept**			**MR-PRESSO global test**	
		**Intercept**	**SE**	***P*-value-**	**RSS_{obs}**	***P*-value**
PD	UC	-0.001	0.006	0.928	56.935	0.274
	CD	0.001	0.006	0.890	64.094	0.144
	IBD	-0.003	0.005	0.568	57.995	0.171
AD	UC	0.001	0.008	0.887	21.141	0.482
	CD	-0.003	0.007	0.660	18.184	0.687
	IBD	-0.001	0.006	0.930	8.924	0.978
