# Supplementary figures and images for: Are neurodegenerative diseases associated with an increased risk of inflammatory bowel disease? A two-sample Mendelian randomization study
Source: Front Immunol. 2022 Sep 8;13:956005. doi: 10.3389/fimmu.2022.956005 (PMC9493012; doi:10.3389/fimmu.2022.956005)

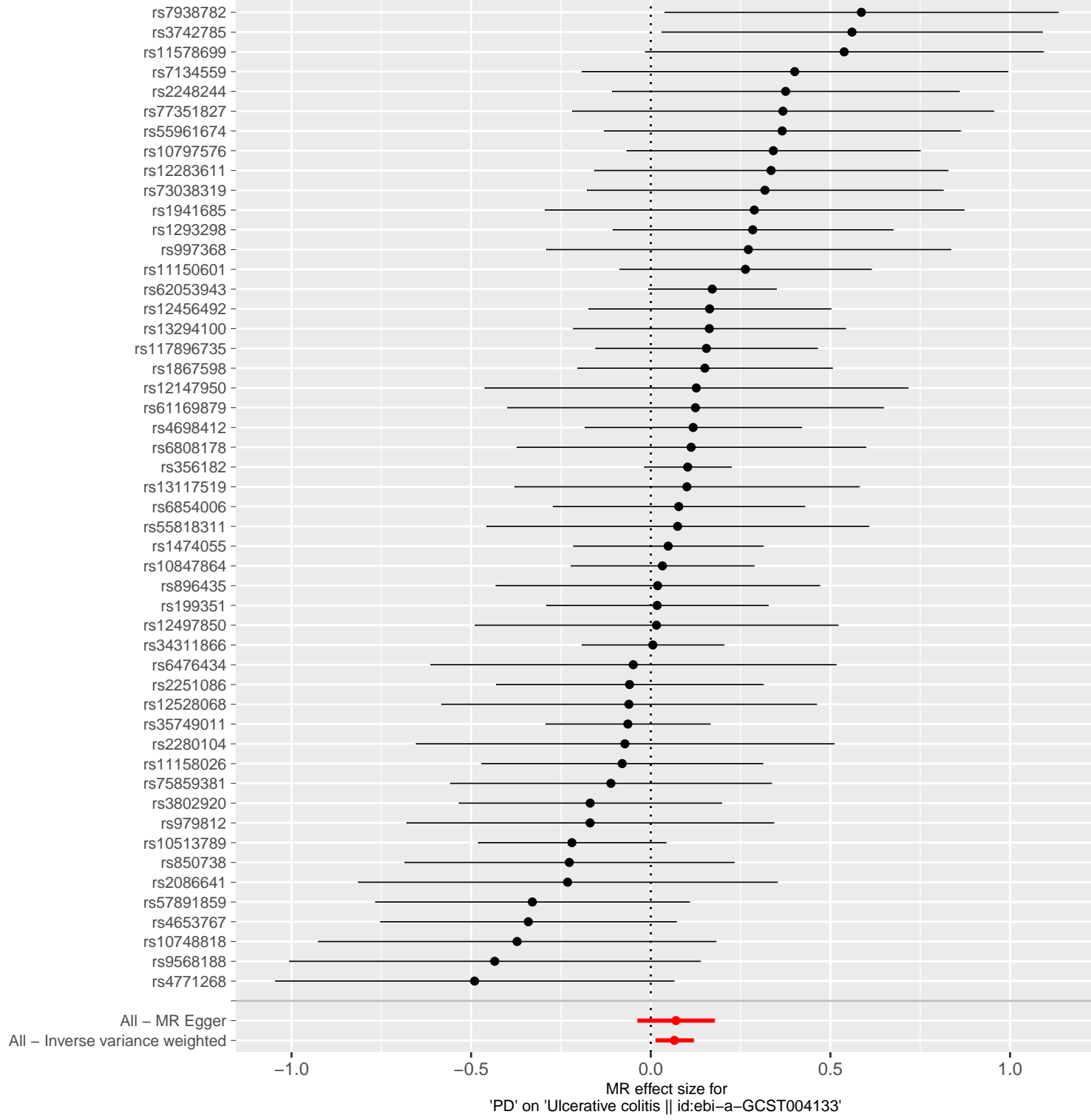

Supplement: Supplementary file 2 [file DataSheet_2.zip › Supplementary Figure/Figure S1. Forest plot for MR analyses of PD on UC.pdf]

# MR Method

- Inverse variance weighted
- MR Egger

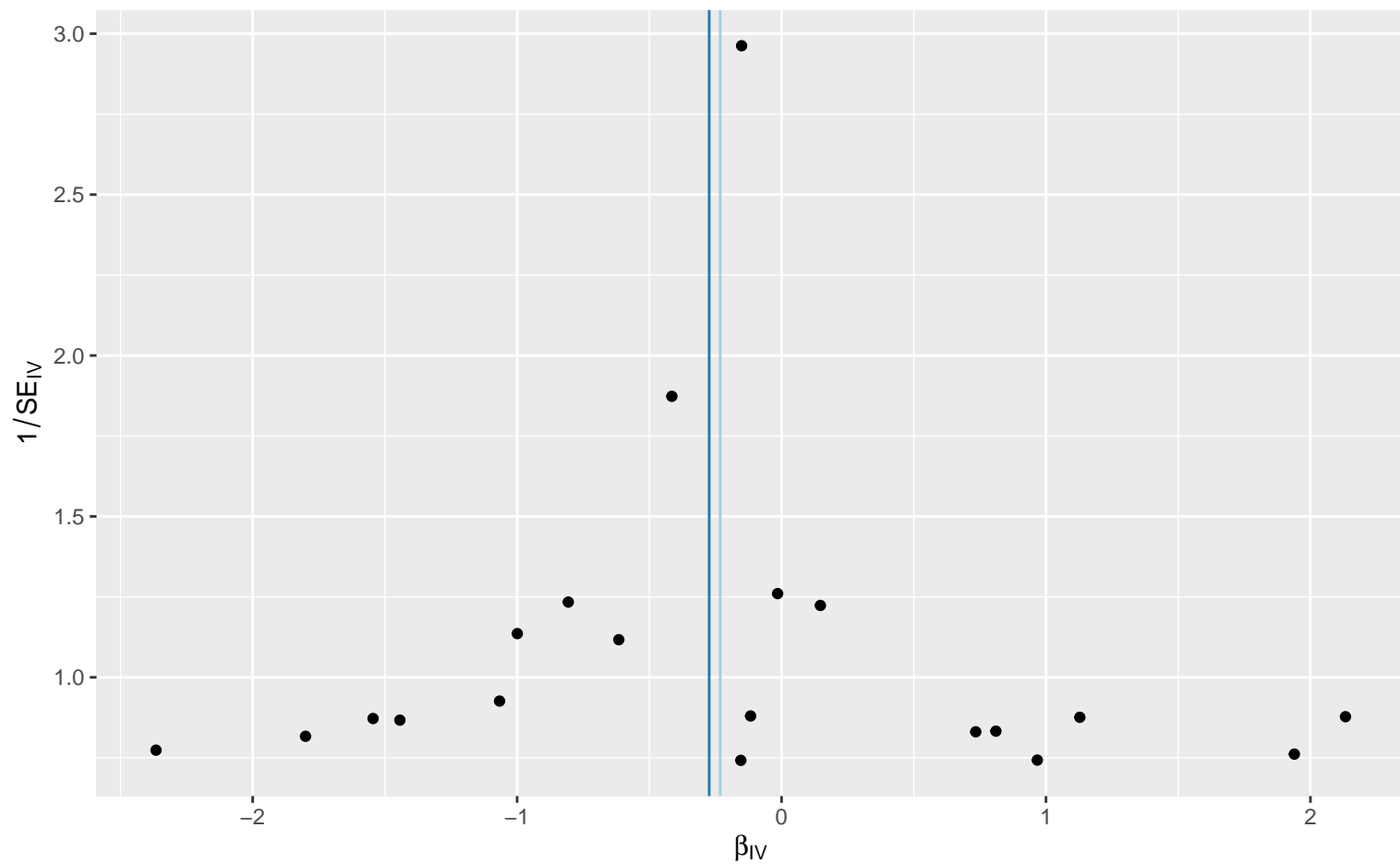

Supplement: Supplementary file 2 [file DataSheet_2.zip › Supplementary Figure/Figure S10. Funnel plot for MR analyses of AD on UC.pdf]

# MR Method

- Inverse variance weighted
- MR Egger

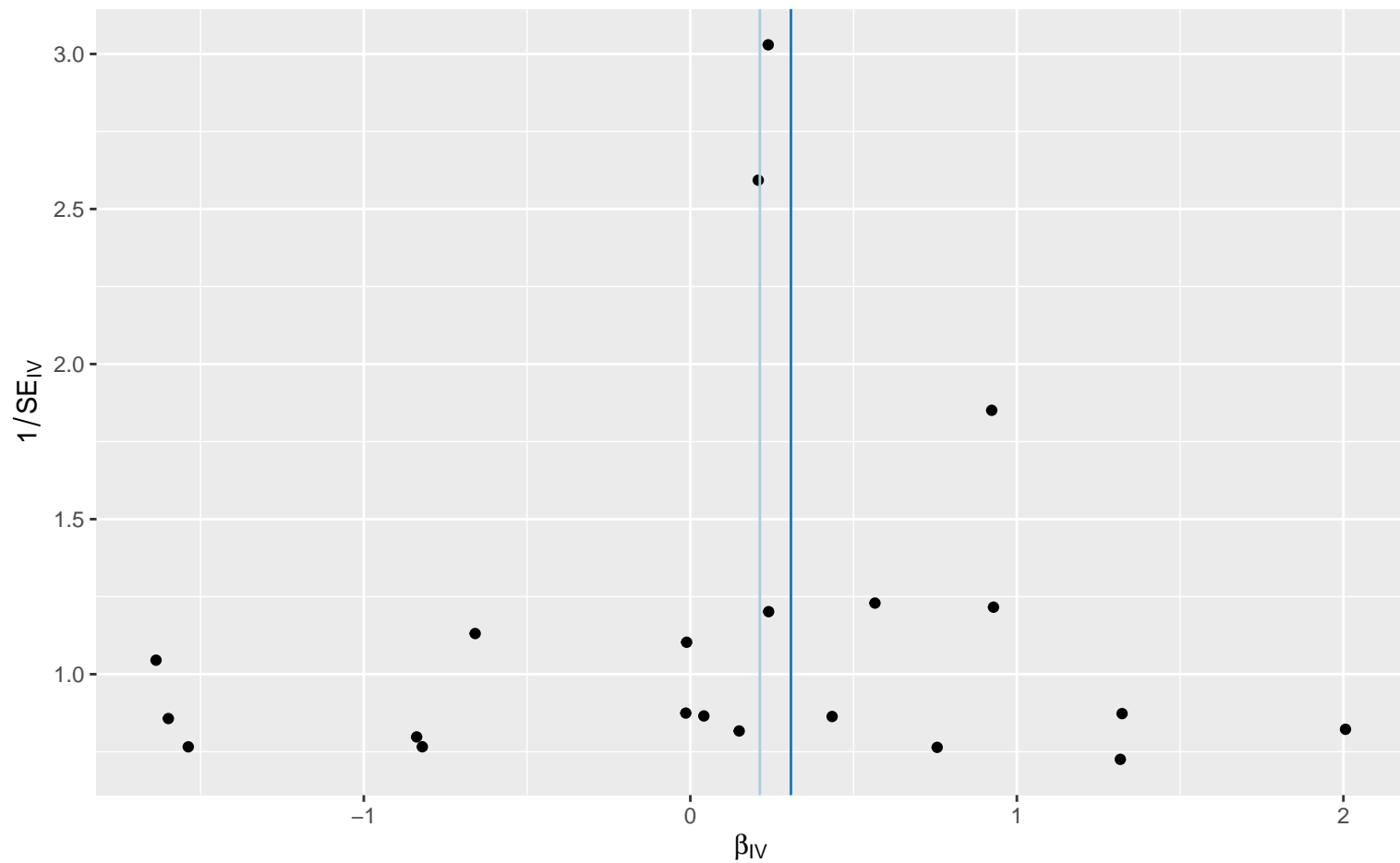

Supplement: Supplementary file 2 [file DataSheet_2.zip › Supplementary Figure/Figure S11. Funnel plot for MR analyses of AD on CD.pdf]

# MR Method

- Inverse variance weighted
- MR Egger

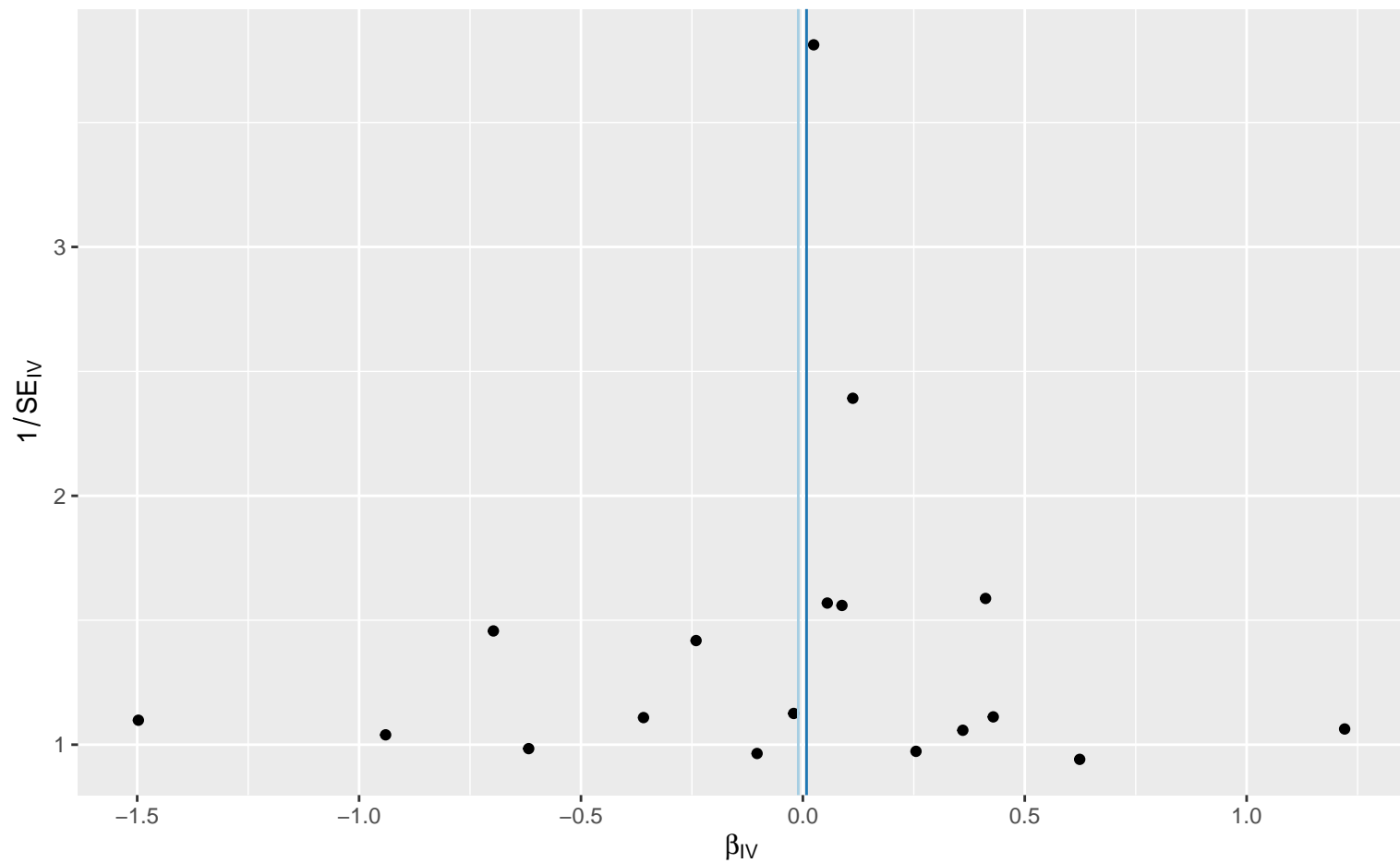

Supplement: Supplementary file 2 [file DataSheet_2.zip › Supplementary Figure/Figure S12. Funnel plot for MR analyses of AD on IBD.pdf]

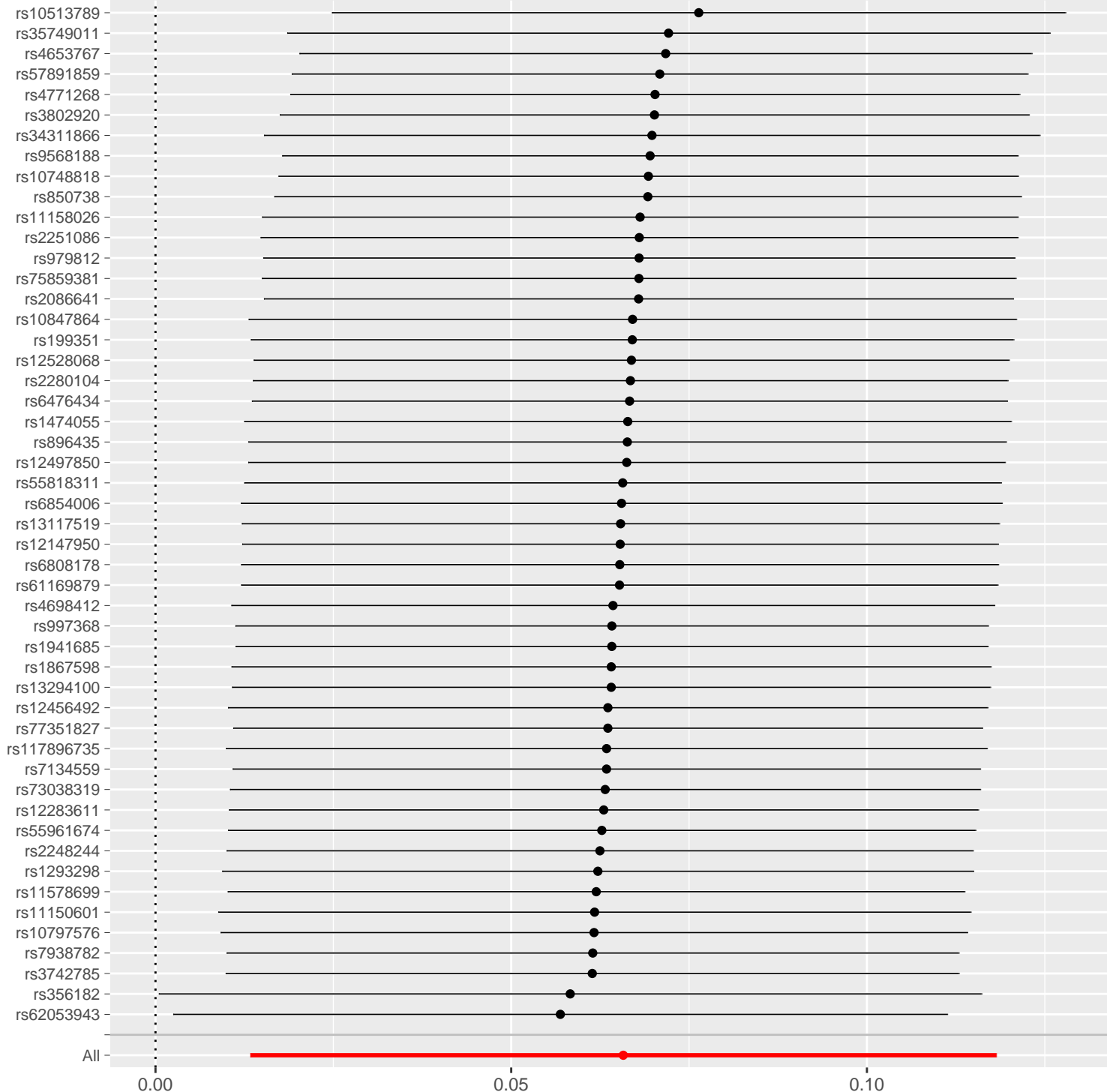

Supplement: Supplementary file 2 [file DataSheet_2.zip › Supplementary Figure/Figure S13. Plot of í░leave-one-outí▒ analyses for MR analyses of PD on UC.pdf]

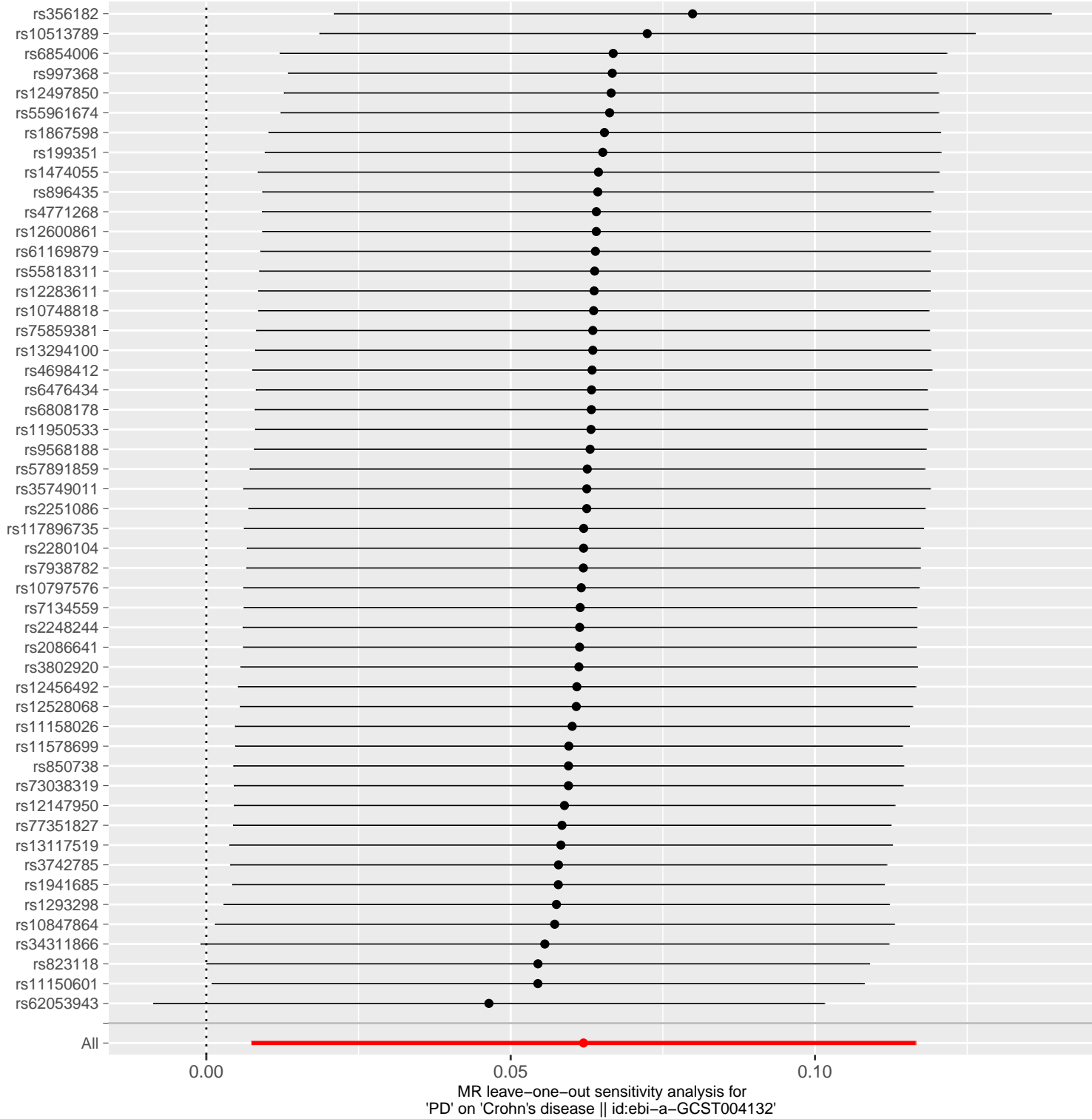

Supplement: Supplementary file 2 [file DataSheet_2.zip › Supplementary Figure/Figure S14. Plot of í░leave-one-outí▒ analyses for MR analyses of PD on CD.pdf]

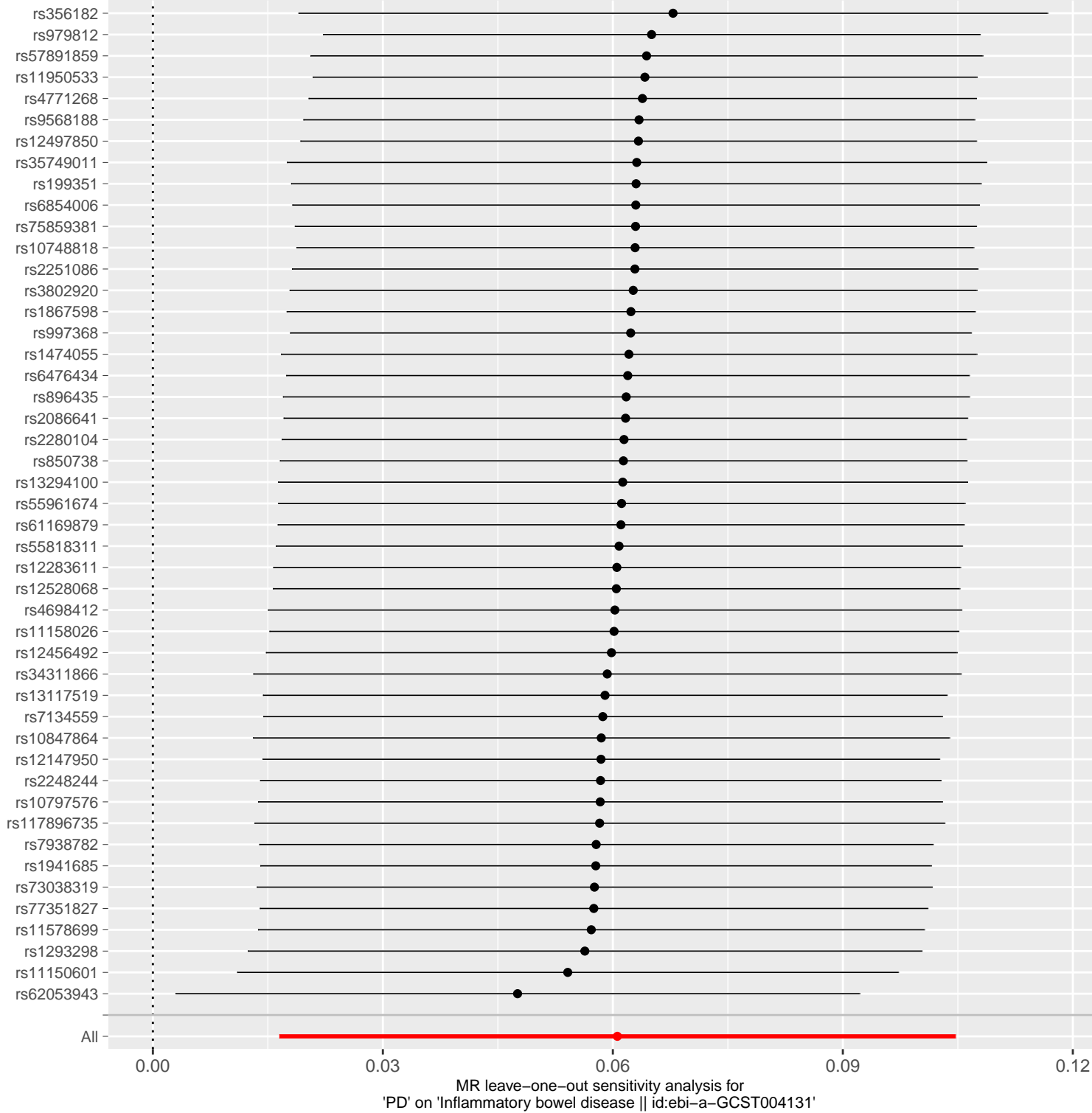

Supplement: Supplementary file 2 [file DataSheet_2.zip › Supplementary Figure/Figure S15. Plot of í░leave-one-outí▒ analyses for MR analyses of PD on IBD.pdf]

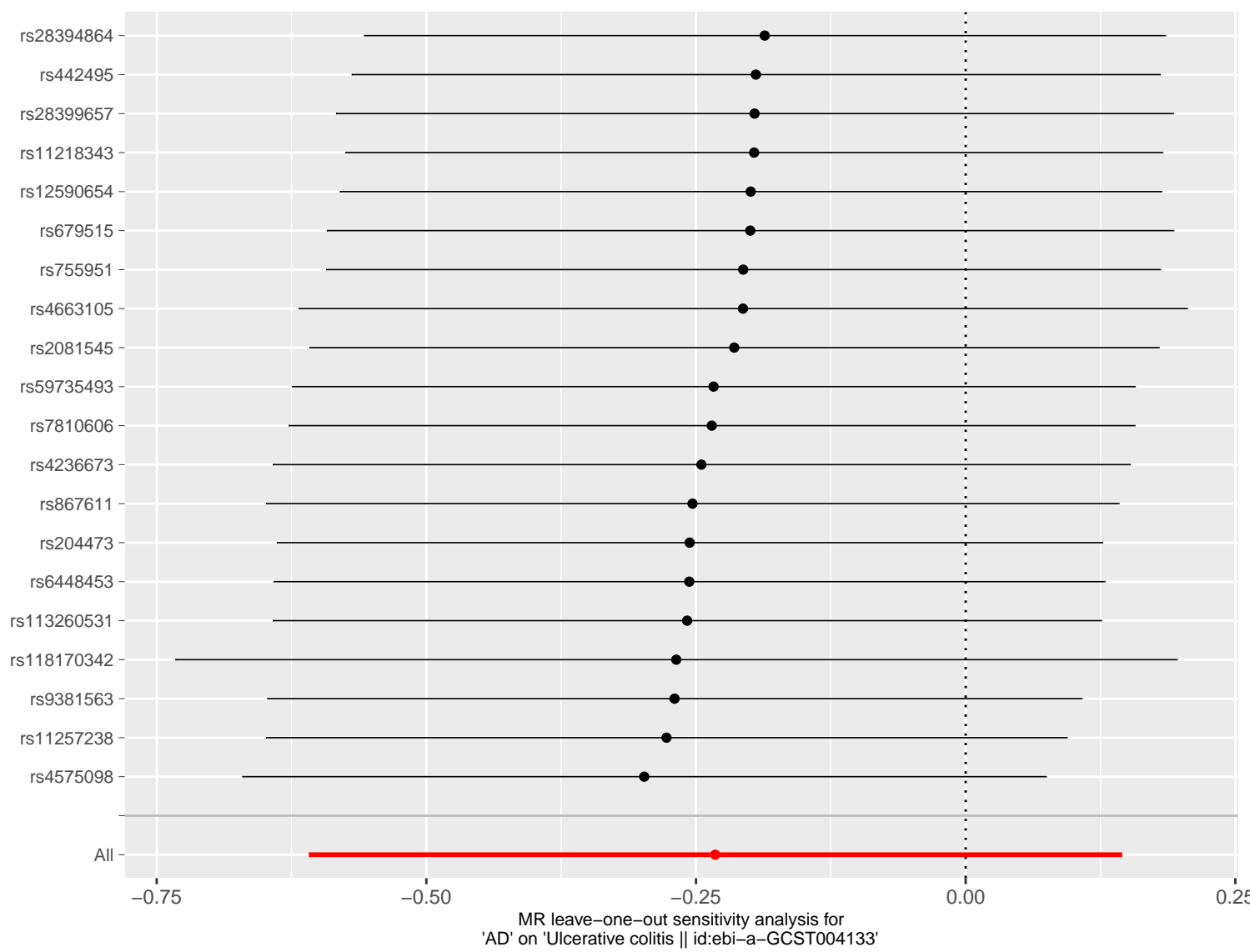

Supplement: Supplementary file 2 [file DataSheet_2.zip › Supplementary Figure/Figure S16. Plot of í░leave-one-outí▒ analyses for MR analyses of AD on UC.pdf]

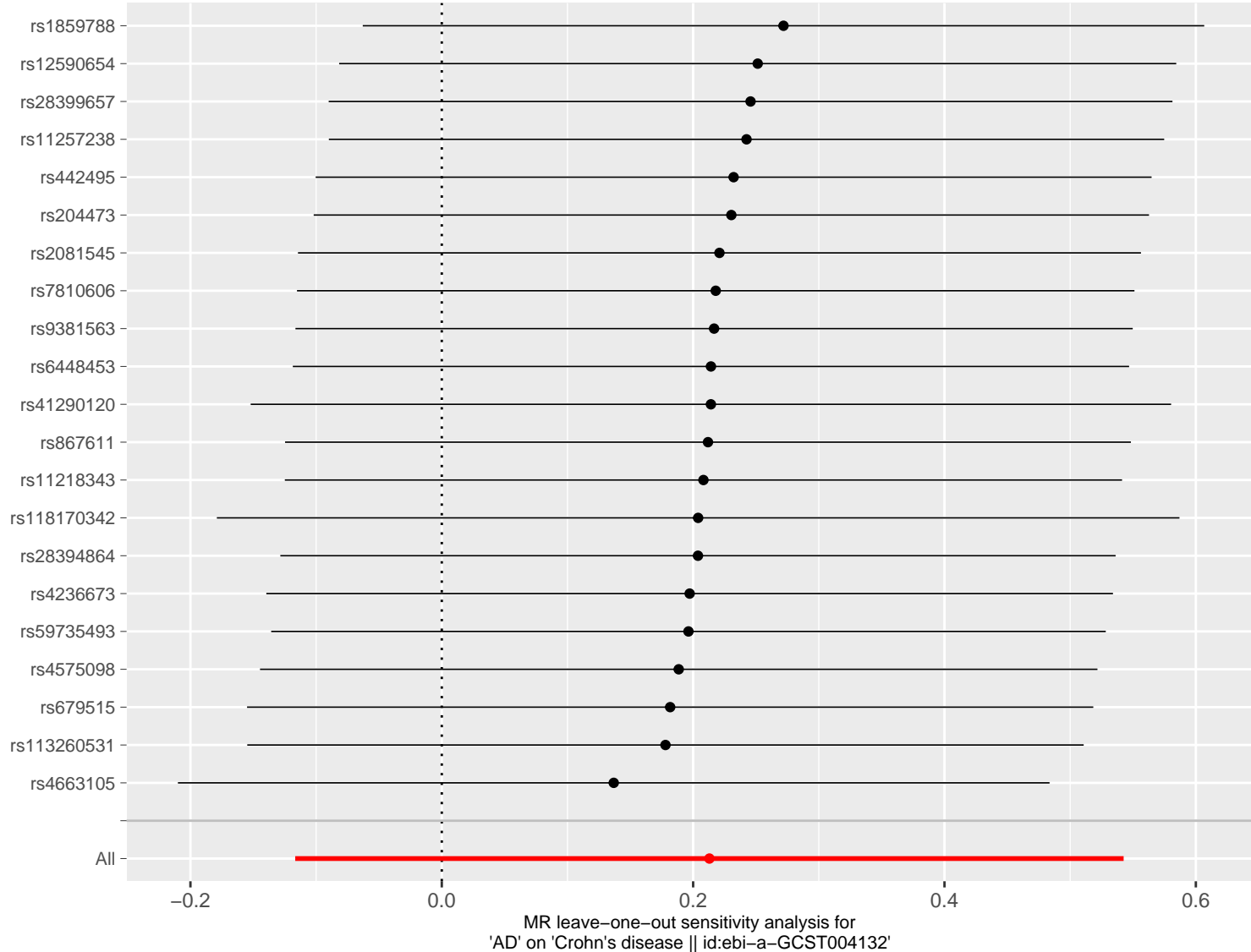

Supplement: Supplementary file 2 [file DataSheet_2.zip › Supplementary Figure/Figure S17. Plot of í░leave-one-outí▒ analyses for MR analyses of AD on CD.pdf]

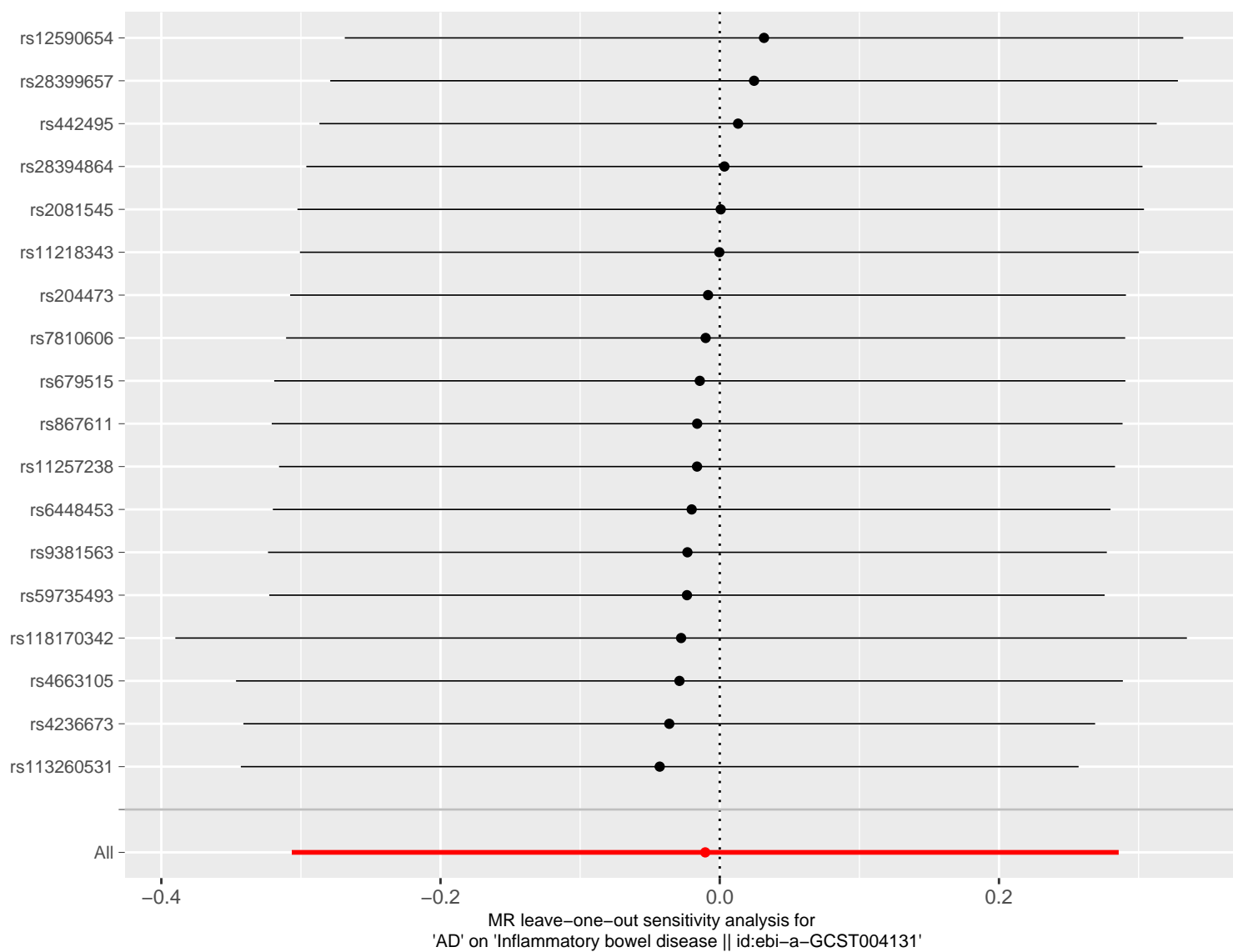

Supplement: Supplementary file 2 [file DataSheet_2.zip › Supplementary Figure/Figure S18. Plot of í░leave-one-outí▒ analyses for MR analyses of AD on IBD.pdf]

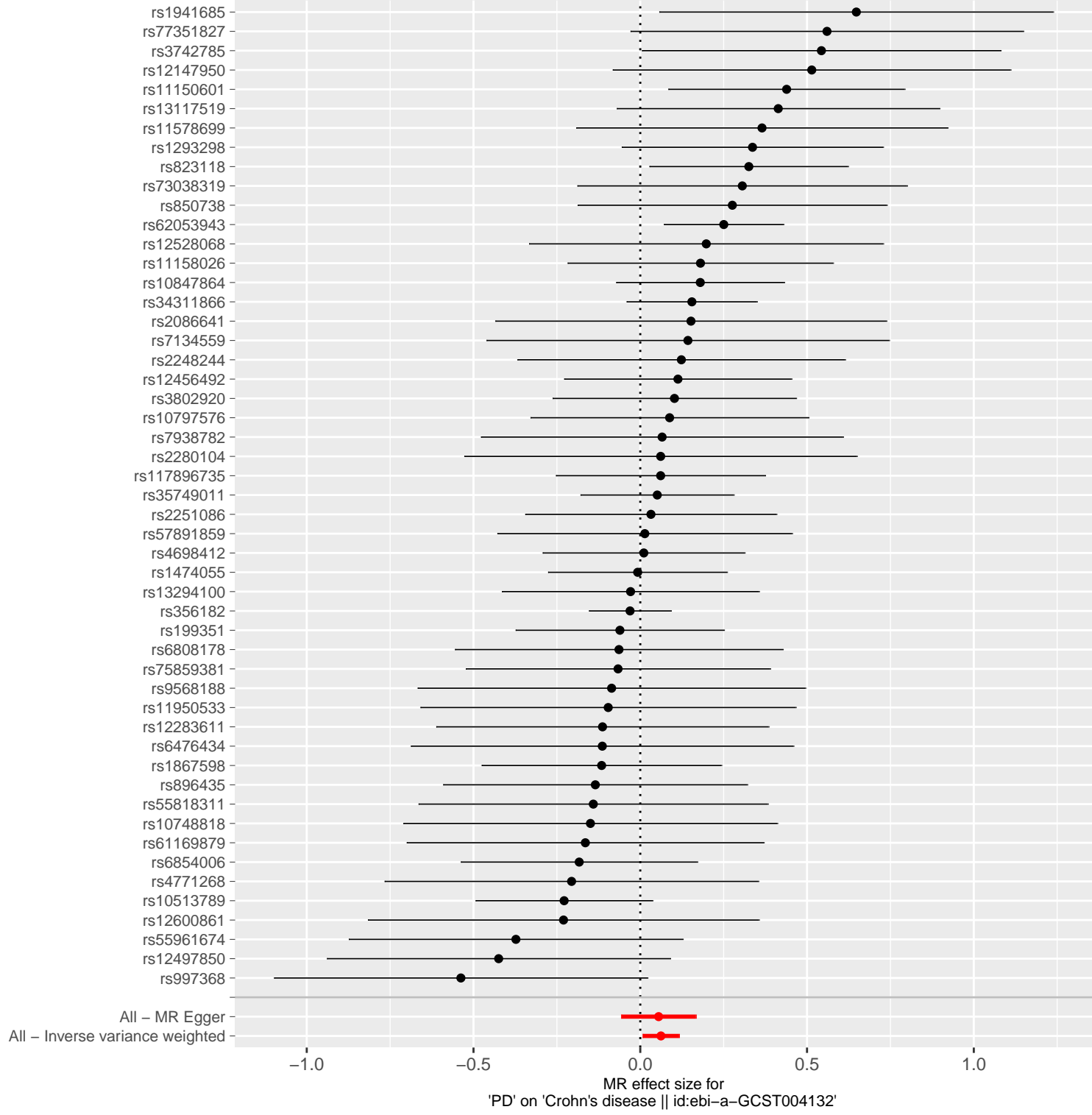

Supplement: Supplementary file 2 [file DataSheet_2.zip › Supplementary Figure/Figure S2. Forest plot for MR analyses of PD on CD.pdf]

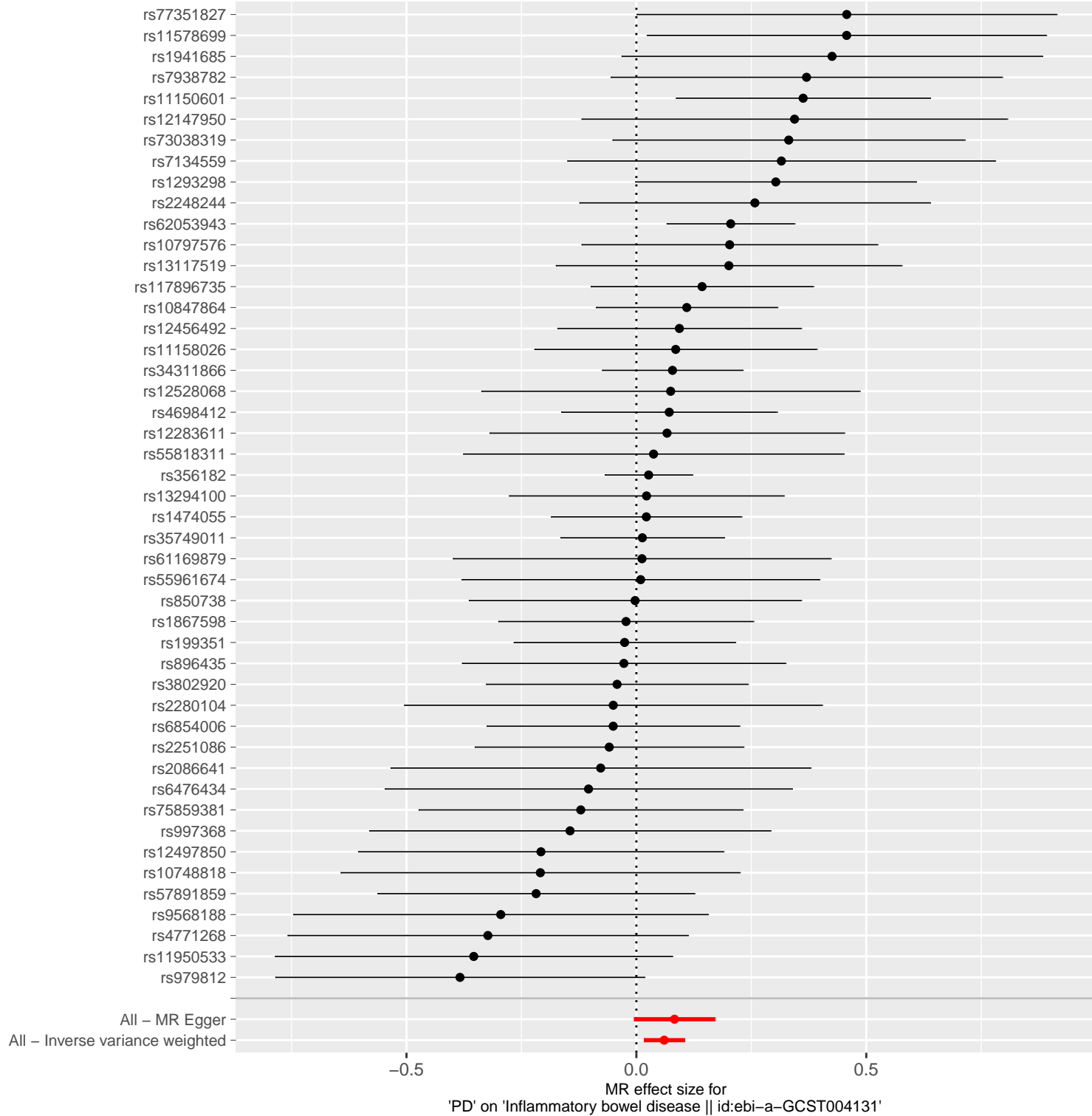

Supplement: Supplementary file 2 [file DataSheet_2.zip › Supplementary Figure/Figure S3. Forest plot for MR analyses of PD on IBD.pdf]

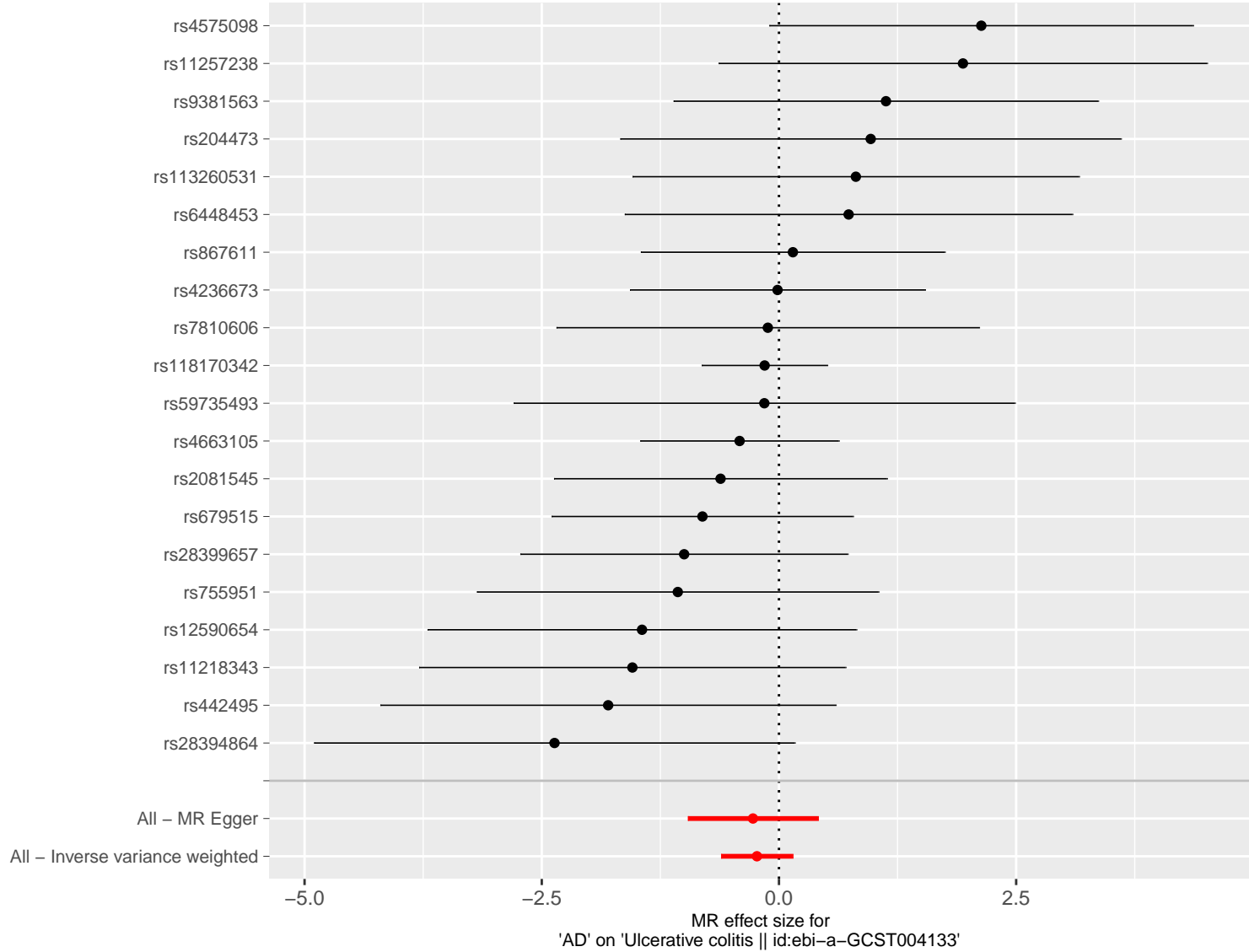

Supplement: Supplementary file 2 [file DataSheet_2.zip › Supplementary Figure/Figure S4. Forest plot for MR analyses of AD on UC.pdf]

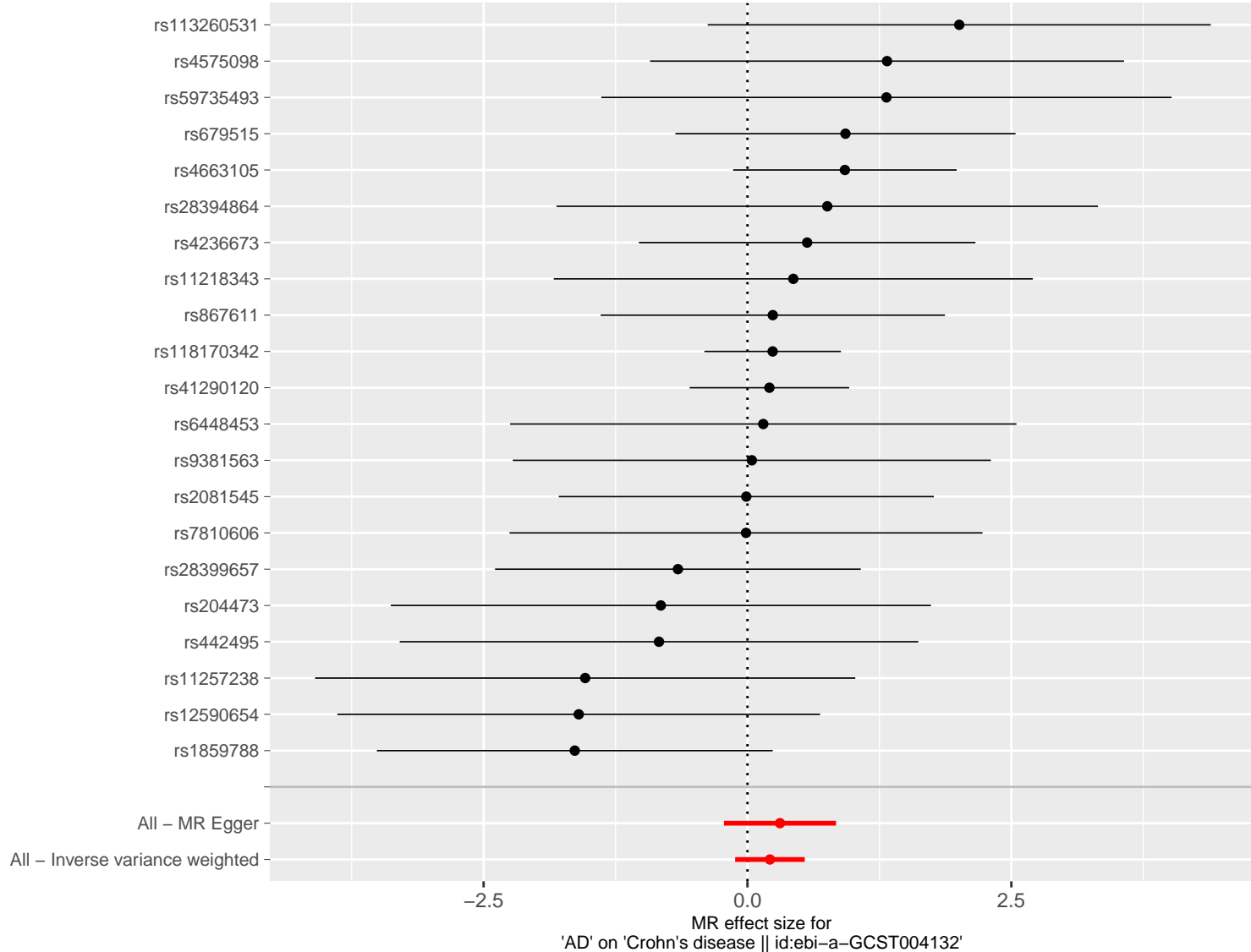

Supplement: Supplementary file 2 [file DataSheet_2.zip › Supplementary Figure/Figure S5. Forest plot for MR analyses of AD on CD.pdf]

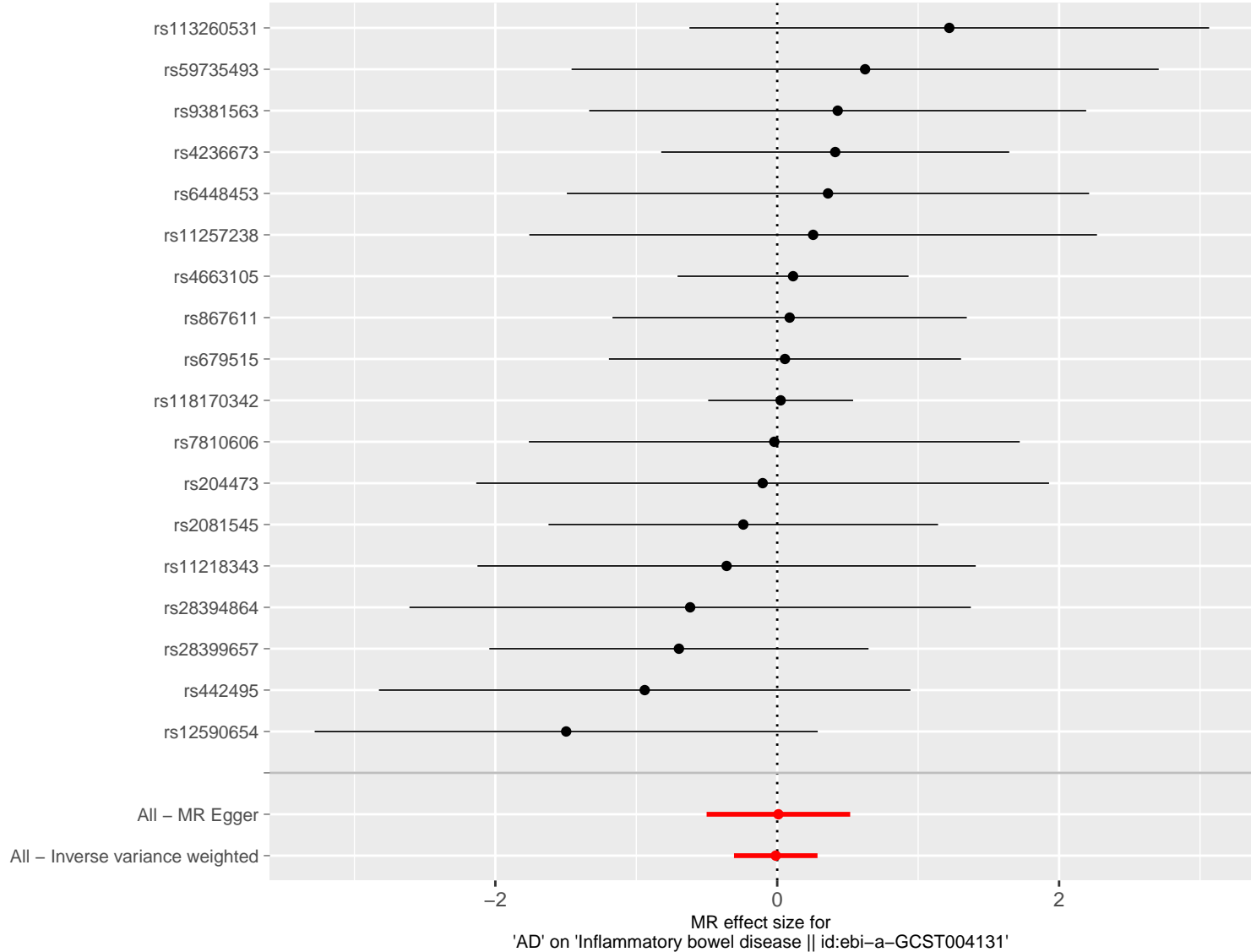

Supplement: Supplementary file 2 [file DataSheet_2.zip › Supplementary Figure/Figure S6. Forest plot for MR analyses of AD on IBD.pdf]

# MR Method

- Inverse variance weighted
- MR Egger

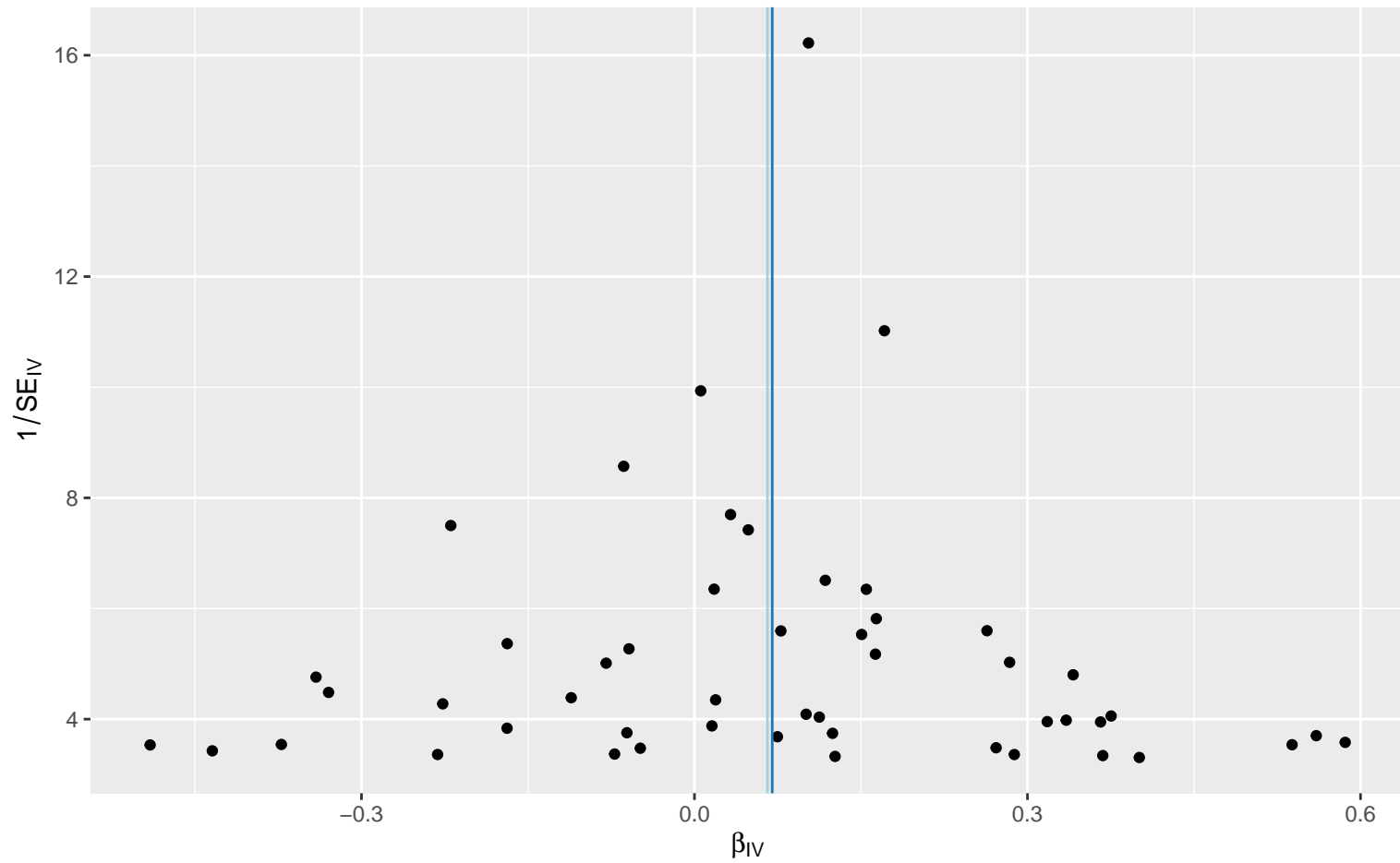

Supplement: Supplementary file 2 [file DataSheet_2.zip › Supplementary Figure/Figure S7. Funnel plot for MR analyses of PD on UC.pdf]

# MR Method

- Inverse variance weighted
- MR Egger

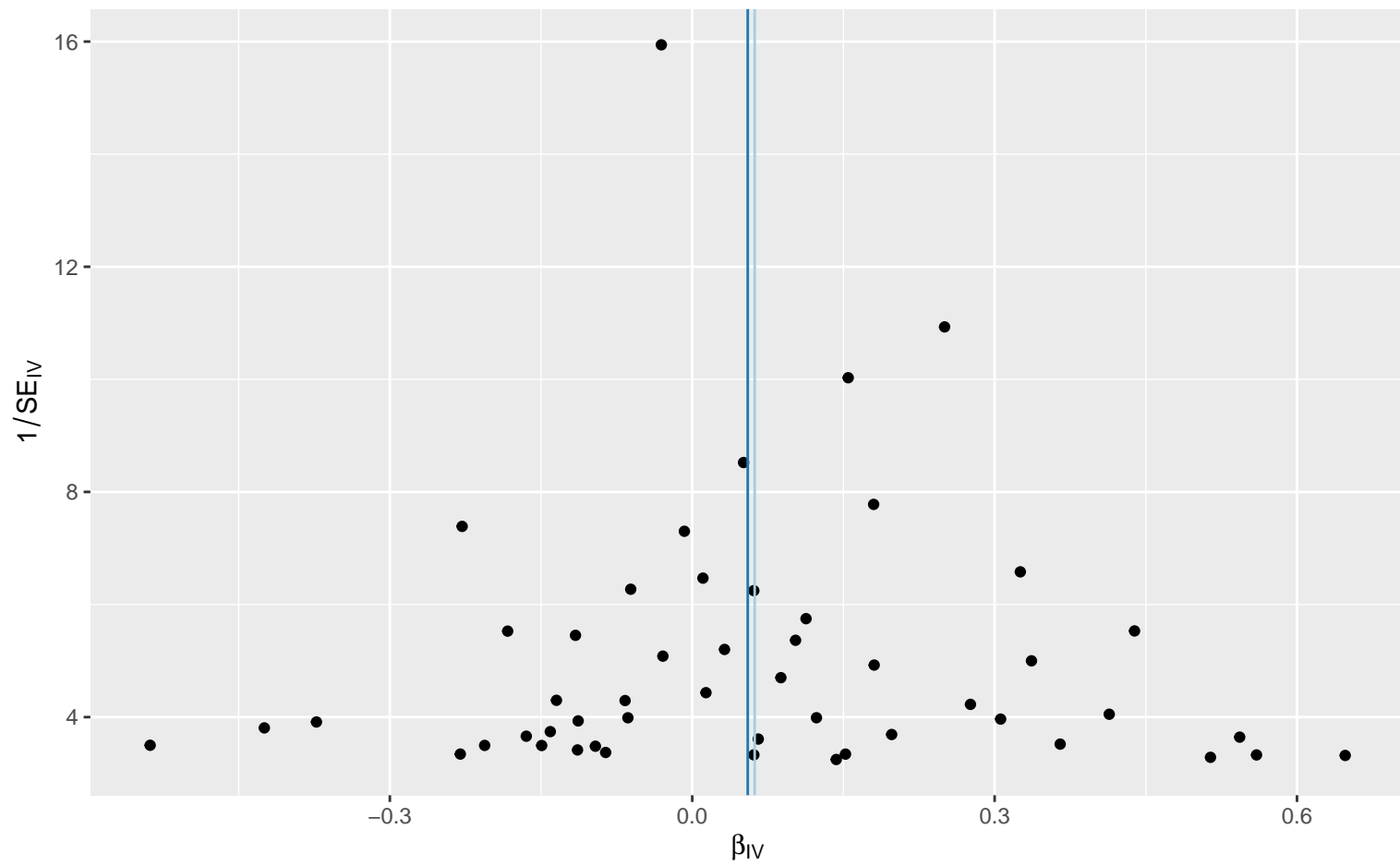

Supplement: Supplementary file 2 [file DataSheet_2.zip › Supplementary Figure/Figure S8. Funnel plot for MR analyses of PD on CD.pdf]

# MR Method

- Inverse variance weighted
- MR Egger

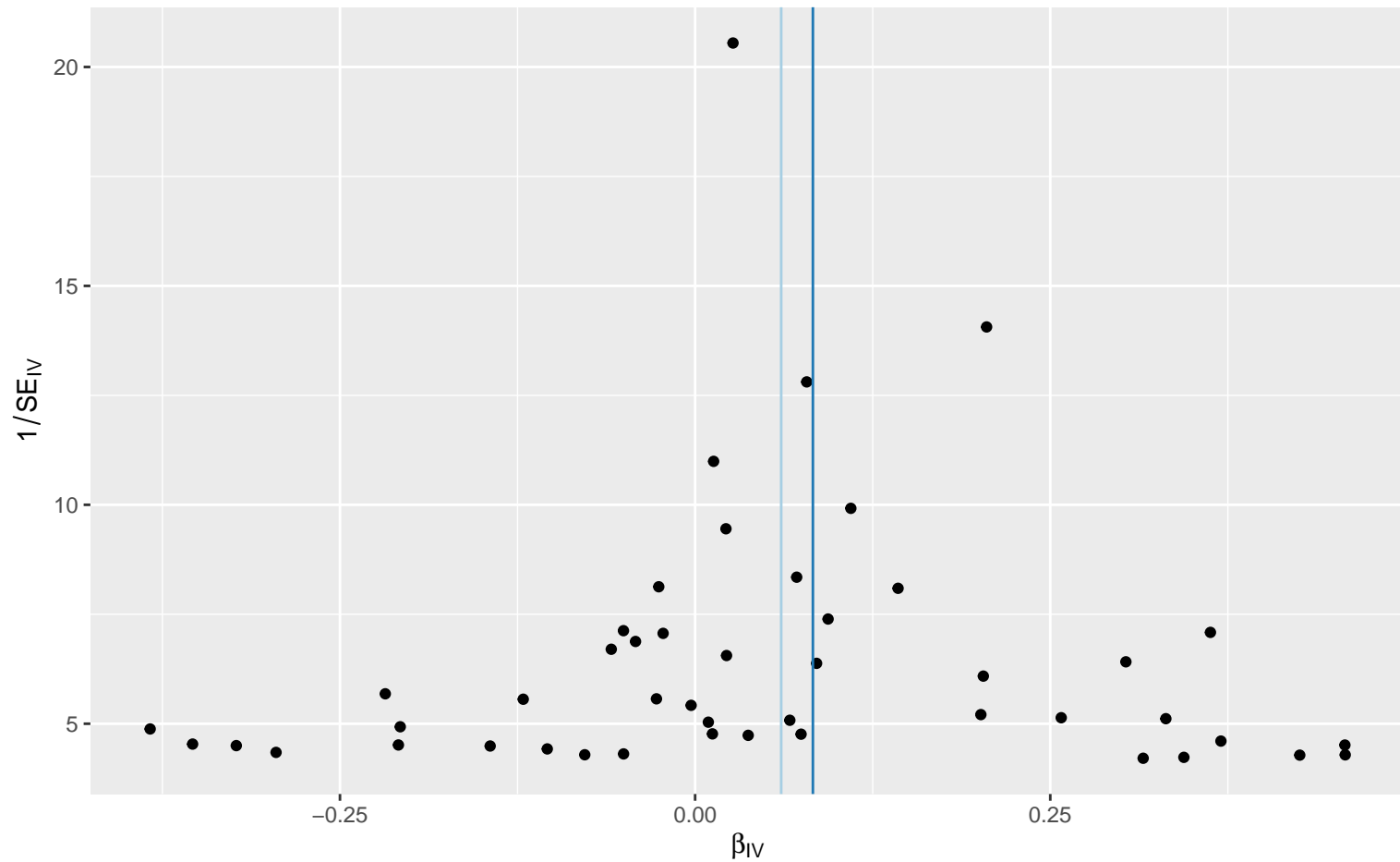

Supplement: Supplementary file 2 [file DataSheet_2.zip › Supplementary Figure/Figure S9. Funnel plot for MR analyses of PD on IBD.pdf]
